# NCLX Inhibition plus Ca^2+^ Delivery Potentiates Electroporation and Antitumor Immunity in Pancreatic Ductal Adenocarcinoma

**DOI:** 10.1002/advs.78288

**Published:** 2026-10-25

**Authors:** Chuanbing Zhao, Long He, Yu Lu, Zanglong Deng, Hongzhen Wei, Yunpeng Zhang, Ming Yang, Tao Yin

**Affiliations:** ^1^ Department of Pancreatic Surgery Union Hospital Tongji Medical College Huazhong University of Science and Technology Wuhan China; ^2^ Sino‐German Laboratory of Personalized Medicine for Pancreatic Cancer Union Hospital Tongji Medical College Huazhong University of Science and Technology Wuhan China; ^3^ Department of Hepato‐Biliary‐Pancreatic Surgery Sichuan Clinical Research Center Sichuan Cancer Center Sichuan Cancer Hospital & Institute Affiliated Cancer Hospital of University of Electronic Science and Technology of China Chengdu China

**Keywords:** antitumor immunity, electroporation, immunogenic pyroptosis, mitochondrial Ca^2+^ overload, NCLX, pancreatic ductal adenocarcinoma

## Abstract

Irreversible electroporation (IRE) is increasingly used for locally advanced pancreatic ductal adenocarcinoma (PDAC), yet electric‐field heterogeneity leaves a low‐field rim where residual cells survive, and recurrence is frequent, compounded by a profoundly immunosuppressive tumor microenvironment. Here, an electroporation‐synchronized adjuvant strategy is developed that integrates intratumoral Ca^2+^ delivery with nanoparticle‐mediated inhibition of mitochondrial NCLX using CGP37157 (mPEG–PCL@CGP37157). This regimen markedly enhances cytotoxicity in PDAC cell lines and patient‐derived organoids and significantly improves local control and survival in syngeneic subcutaneous and orthotopic PDAC models, without overt systemic toxicity. Reduced therapeutic efficacy in T cell–deficient hosts demonstrates the importance of adaptive immunity. Mechanistically, EP‐resistant PDAC cells upregulate NCLX to buffer mitochondrial Ca^2+^ stress. Concurrent Ca^2+^ delivery and pharmacological NCLX inhibition induce sustained mitochondrial Ca^2+^ overload, triggering immunogenic GSDMD‐dependent pyroptosis. This is accompanied by enhanced dendritic cell activation and CD8^+^ T‐cell responses, as well as suppression of a tumor–myeloid SPP1–CD44 axis, promoting pro‐inflammatory macrophage polarization. Collectively, targeting NCLX potentiates the immunogenic effects of IRE and provides a translatable strategy for eliminating residual disease in PDAC.

## Graphical abstract

Electroporation combined with NCLX inhibition and Ca^2+^ delivery induces mitochondrial Ca^2+^ overload in PDAC cells, triggering pyroptosis, DAMP release, and antitumor immune activation. Simultaneously, suppression of tumor‐derived SPP1 weakens SPP1–CD44 signaling and remodels macrophages toward an antitumor phenotype, thereby enhancing CD8^+^ T‐cell responses and reducing tumor progression and metastasis.

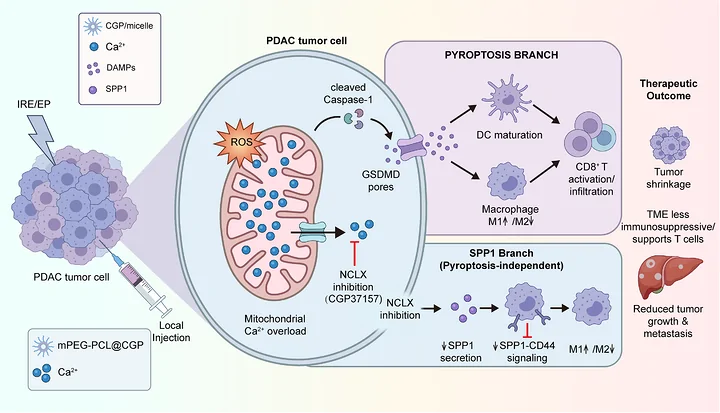

**Abbreviations**
CaCGP: combined local delivery of Ca^2+^ and CGP37157‐loaded micelles
CGP: MPEG‐PCL@CGP37157 Micelles
EP: electroporation
GSEA: gene set enrichment analysis
IRE: irreversible electroporation
PDAC: pancreatic ductal adenocarcinoma
TME: tumor microenvironment

## 1 Introduction

Pancreatic ductal adenocarcinoma (PDAC) is among the most aggressive malignancies of the digestive system, characterized by an extremely poor prognosis and continually rising incidence and mortality rates [1]. The overall 5‐year survival rate remains approximately 13% [2]. At diagnosis, PDAC is generally classified according to anatomical resectability and disease extent into resectable, borderline resectable, locally advanced, and metastatic disease [3]. Resectable PDAC is typically defined by the absence of major arterial involvement and absent or reconstructable venous involvement, and surgical resection followed by adjuvant chemotherapy remains the principal curative‐intent treatment strategy. Borderline resectable PDAC is characterized by limited vascular involvement and often requires neoadjuvant therapy followed by reassessment for surgical feasibility. In contrast, locally advanced PDAC frequently presents with extensive vascular encasement that precludes upfront resection, and its management mainly relies on induction chemotherapy, chemoradiotherapy, and selected local treatment approaches, aiming to achieve disease control, symptom relief, and potential conversion to resection. However, even after induction therapy, the conversion‐to‐resection rate for locally advanced PDAC remains limited [4, 5]. Therefore, more effective local therapeutic strategies are urgently needed to complement and synergize with systemic treatment and improve patient outcomes.

Irreversible electroporation (IRE) is a non‐thermal local treatment modality that applies short, high‐voltage electrical pulses to generate irreversible nanopores in the tumor cell membrane, thereby disrupting cellular homeostasis and inducing cell death [6]. Clinical studies have shown that IRE can improve survival outcomes in patients with locally advanced PDAC and can alleviate cancer‐related pain while improving quality of life [7, 8]. Accordingly, it has been approved for the treatment of locally advanced PDAC. However, the intrinsic heterogeneity of electrical conductivity within tumor tissue often leads to an uneven electric‐field distribution, resulting in a “reversible electroporation (RE)” zone at the ablation margin. Residual tumor cells within this RE zone can survive and rapidly repopulate, constituting a major source of incomplete ablation and early recurrence [9, 10]. Notably, the molecular basis by which residual tumor cells adapt to electroporation stress and persist within the RE zone remains unclear, which constrains the development of mechanism‐guided interventions to prevent recurrence. In parallel, the immunosuppressive tumor microenvironment (TME), a defining feature of PDAC, represents another major barrier to therapeutic improvement [11]. Although IRE can elicit a degree of immune activation, the response is typically insufficient to establish a potent and durable antitumor immunity capable of eradicating minimal residual disease or preventing recurrence and distant progression [12, 13, 14]. Therefore, enhancing local tumor clearance while overcoming immunosuppression to induce systemic antitumor immunity remains a central scientific and translational challenge for improving IRE efficacy.

From the “seed–soil” perspective, tumor recurrence is determined not only by residual tumor cells themselves (the “seed”), but also by an immunosuppressive microenvironment that supports their regrowth (the “soil”) [15, 16, 17]. Accordingly, failure of IRE/electroporation‐based therapy may arise from two coupled limitations: on the one hand, residual tumor cells at the ablation margin acquire adaptive programs that enable survival and regrowth; on the other hand, the post‐treatment TME can maintain or re‐establish immunosuppressive networks, thereby providing a permissive niche for recurrence. Notably, the shared regulatory nodes linking residual cell survival (seed) and immunosuppressive TME maintenance (soil) after electroporation (EP) remain elusive. Mitochondrial Ca^2+^ homeostasis, a core adaptive mechanism for cell survival, has been shown to modulate immunogenic cell death and myeloid cell function, suggesting that it may serve as a mechanistic link between these processes; however, its upstream regulators in PDAC EP resistance remain unknown.

Previous studies suggest that although EP can directly damage the plasma membrane, tumor cells exposed to sublethal field strengths may still restore viability through homeostatic repair programs, a phenomenon that is particularly pronounced at the ablation margin [18]. Accumulating evidence further indicates that mitochondria represent a critical hub in this adaptive process. Electroporation‐induced ionic imbalance can profoundly disrupt mitochondrial calcium (Ca^2+^) homeostasis, and mitochondrial Ca^2+^ overload is considered a key driver of lethal injury [19, 20]. In the dynamic regulation of mitochondrial Ca^2+^, the mitochondrial Na^+^/Ca^2+^ exchanger (NCLX) serves as the major efflux pathway [21]. As the major mitochondrial Ca^2+^ efflux exchanger, NCLX has been implicated in tumor cell survival under metabolic stress in breast and lung cancer, but its role in PDAC resistance to electroporation and crosstalk with the immunosuppressive TME remains completely unaddressed. It is therefore plausible that residual tumor cells may increase Ca^2+^ extrusion to rapidly mitigate mitochondrial Ca^2+^ burden, thereby raising the resistance threshold to electroporation‐induced Ca^2+^ stress and promoting survival, resistance, and recurrence. Conversely, mitochondrial Ca^2+^ status may also shape post‐ablation immune remodeling by influencing cell‐death modality and its immunological consequences (e.g., inflammatory and immunogenic cell death). Collectively, interventions that constrain mitochondrial Ca^2+^ efflux may simultaneously enhance elimination of residual tumor cells (seed) and alleviate immunosuppressive microenvironmental features (soil).

Here, we identify NCLX upregulation as a key adaptive mechanism that supports PDAC cell survival following electroporation‐induced stress. Accordingly, we develop an EP+CaCGP strategy that integrates electroporation, NCLX inhibition, and exogenous Ca^2+^ supplementation to induce sustained mitochondrial Ca^2+^ overload. We further demonstrate that this approach enhances therapeutic efficacy by promoting immunogenic pyroptotic cell death and suppressing SPP1–CD44‐mediated tumor–macrophage crosstalk. Collectively, these findings establish NCLX as a targetable vulnerability for improving electroporation‐based therapies and for reprogramming the immunosuppressive PDAC microenvironment.

## 2 Results

### 2.1 EP Induces Mitochondrial Damage via Mitochondrial Ca^2+^ Overload

To investigate the impact of IRE on PDAC, we established a murine orthotopic PDAC model and performed IRE treatment. Survival analysis showed that, compared with the control group, IRE treatment did not significantly prolong the survival of mice (Figure 1A). Hematoxylin and eosin (H&E) staining revealed that a small number of cancer cells remained viable after IRE treatment (Figure 1B), and the proliferation rate of these surviving tumor cells increased over time (Figure 1C,D).

**FIGURE 1:**
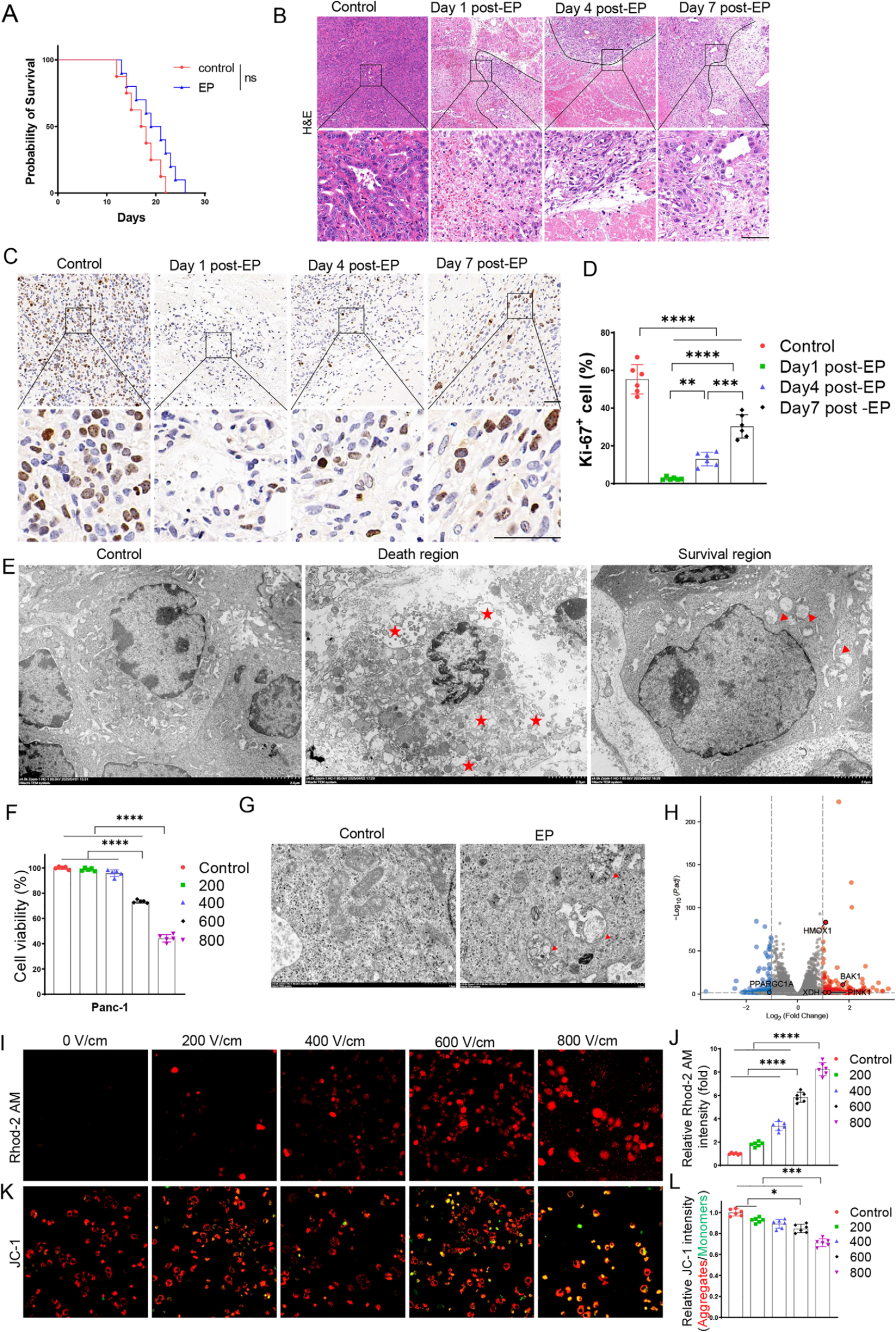
Electroporation induces mitochondrial injury that correlates with mitochondrial Ca^2+^ accumulation in pancreatic cancer. (A) Kaplan–Meier survival analysis of mice bearing orthotopic pancreatic tumors treated with sham or electroporation (EP). Control group: n = 8; IRE group: n = 10. (B) Representative H&E staining of orthotopic pancreatic tumors before and at day 1, day 4, and day 7 after EP, showing ablated regions and residual viable tumor cells. (scale bar, 50 µm). n = 6 per group. (C,D) Representative Ki‐67 staining and quantification of Ki‐67^+^ tumor cells at day 1, day 4, and day 7 after EP, indicating progressive proliferation of residual tumor cells. (scale bar, 50 µm). n = 6 per group. (E) Transmission electron microscopy (TEM) of tumor tissues after EP showing severe mitochondrial disruption in the ablated (death) region and comparatively mild mitochondrial injury in the surviving region (scale bar, 2.0 µm). n = 6 per group. (F) Cell viability of Panc‐1 cells after electroporation (EP) at the indicated electric‐field strengths. n = 5 per group. (G) TEM of Panc‐1 cells after EP showing mitochondrial swelling and cristae disruption (scale bar, 1.0 µm). n = 6 per group. (H) Transcriptomic analysis of EP‐treated Panc‐1 cells showing induction of mitochondrial stress/injury–associated genes (e.g., *HMOX1*, *BAK1*, *PINK1*). n = 3 per group. (I,J) Representative images and quantification of Rhod‐2 AM fluorescence indicating increased mitochondrial Ca^2+^ levels with increasing EP field strength. n = 6 per group. (K,L) Representative images and quantification of JC‐1 staining showing loss of mitochondrial membrane potential with increasing EP field strength. n = 6 per group. Data are presented as mean ± s.e.m. Statistical significance was assessed by log‐rank (Mantel–Cox) test (A), two‐tailed Student's *t* test (two groups), or one‐way ANOVA (multiple groups). *p < 0.05; **p < 0.01; ***p < 0.001; ****p < 0.0001. ns, not significant.

Transmission electron microscopy (TEM) of pancreatic tumor tissues after IRE treatment revealed evident mitochondrial damage. In the dead/ablated regions, cells exhibited complete mitochondrial disintegration. However, in the surviving regions, tumor cells displayed only mild mitochondrial injury, such as outer membrane swelling, matrix rarefaction, and loss of cristae (Figure 1E). Interestingly, mitochondrial damage in the surviving regions peaked on day 1 after IRE but largely recovered by day 7, suggesting structural repair of mitochondria in the surviving tumor cells (Figure S1A).

To further explore the effects of electroporation (EP) on tumor cells, Panc‐1 and KPC A548 cell lines were exposed to different electric field intensities. At 600 V/cm, cell viability was significantly reduced in both cell lines (Figures 1F and Figure S1B); therefore, this field strength was selected for subsequent in vitro experiments. TEM analysis of EP‐treated Panc‐1 cells confirmed mitochondrial damage, including outer membrane swelling, cristae loss, and fragmentation (Figure 1G). Transcriptomic sequencing of EP‐treated Panc‐1 cells showed upregulation of mitochondrial damage–related genes (such as HMOX1, BAK1, and PINK1) (Figure 1H). Consistently, Gene Set Enrichment Analysis (GSEA) demonstrated significant enrichment of pathways involved in mitochondrial organization and mitochondrial functional homeostasis after EP treatment, suggesting that EP induces a mitochondrial stress response accompanied by mitochondrial dysfunction (Figures S1C,D).

EP‐induced mitochondrial damage is associated with ionic imbalance, particularly elevated intracellular calcium levels [18]. Increasing EP field strength correlated with higher mitochondrial calcium content (Figure 1I,J) and led to a reduction in mitochondrial membrane potential (Figure 1K,L), and similar results were observed in KPC A548 cells (Figure S1E–H). Collectively, EP‐induced tumor cell death is closely associated with mitochondrial damage driven by calcium elevation and ionic imbalance, which affects the therapeutic efficacy of EP‐based treatment.

### 2.2 NCLX Upregulation Promotes EP Resistance in PDAC Cells by Enhancing Mitochondrial Ca^2+^ Efflux

To elucidate the molecular basis underlying PDAC cell adaptation to repeated electroporation stress, we established a relatively EP‐resistant Panc‐1 cell line through repeated EP challenges. Compared with parental cells, these EP‐resistant cells exhibited significantly enhanced survival after EP in the CCK‐8 and colony formation assays, indicating increased resistance to EP‐induced injury rather than complete resistance to electroporation (Figure S2A–C). Under identical EP conditions, EP‐resistant cells displayed significantly lower mitochondrial Ca^2+^ levels (Figure 2A,B) and a substantially attenuated loss of mitochondrial membrane potential (Figure 2C,D), suggesting an improved ability to maintain mitochondrial Ca^2+^ homeostasis and partially resist EP‐induced mitochondrial injury.

**FIGURE 2:**
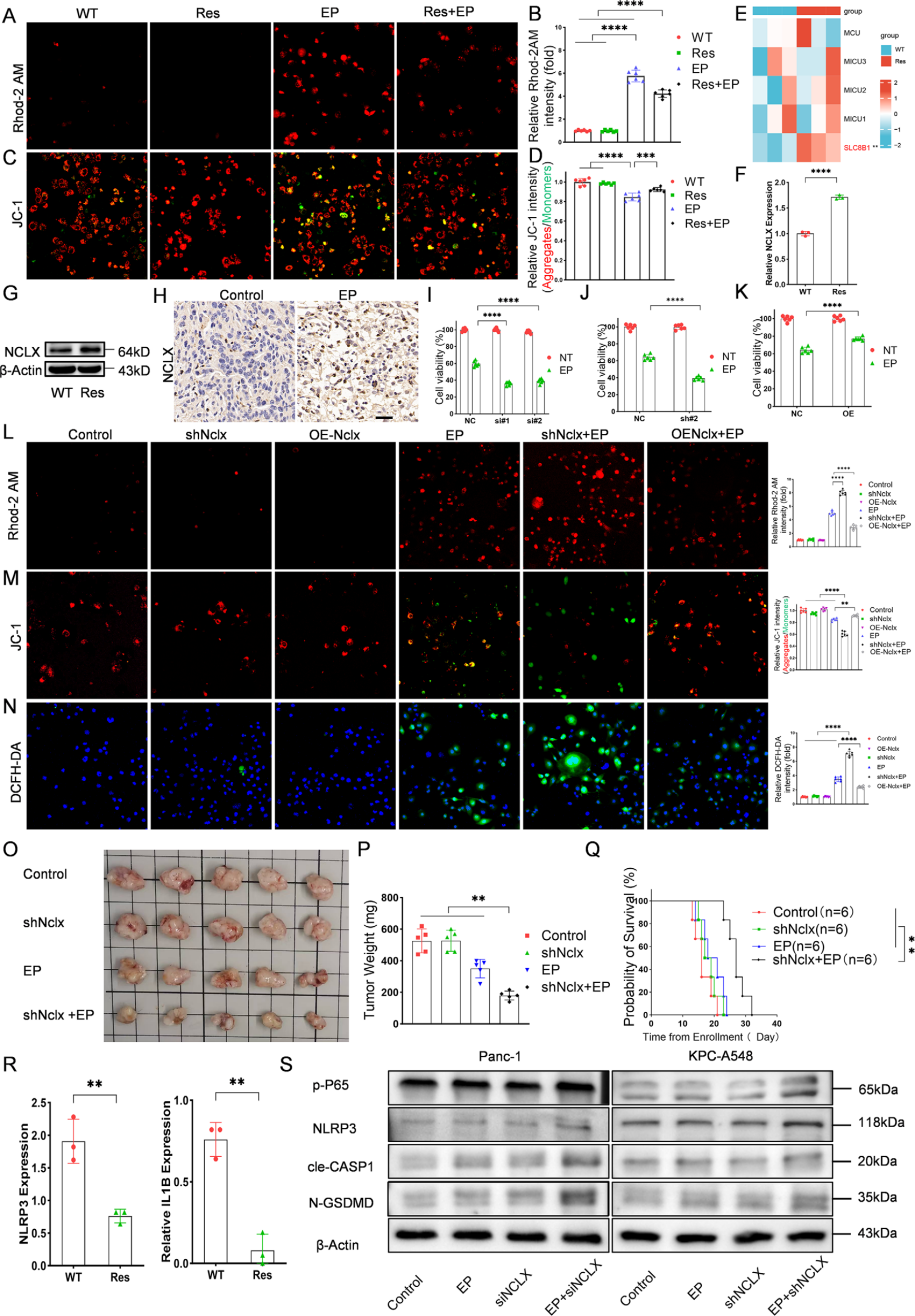
NCLX‐mediated regulation of mitochondrial calcium determines sensitivity to electroporation (EP) in pancreatic cancer. (A, B) Mitochondrial Ca^2+^ levels measured by Rhod‐2 AM fluorescence in wild‐type (WT) and EP‐resistant (Res) Panc‐1 cells (n = 6 per group). (C, D) Mitochondrial membrane potential assessed by JC‐1 staining in the indicated groups (n = 6 per group). (E) Heatmap showing RNA‐seq–based expression profiles of genes regulating mitochondrial calcium channels in WT and EP‐resistant Panc‐1 cells (n = 3 per group). (F) Reverse transcription quantitative polymerase chain reaction (RT‐qPCR) analysis showing the relative expression of SLC8B1 in WT and EP‐resistant Panc‐1 cells (n = 3 per group). (G) Western blot analysis of NCLX protein expression in WT and EP‐resistant Panc‐1 cells (n = 3 per group). (H) Immunohistochemical staining of NCLX in tumor sections after EP treatment (scale bar, 50 µm) (n = 6 per group). (I, J) Cell viability following EP in Panc‐1 and KPC A548 cells with NCLX knockdown (siRNA or lentiviral), compared with corresponding controls (n = 6 per group). (K) Cell viability following EP in NCLX‐overexpressing cells (n = 6 per group). (L) Mitochondrial Ca^2+^ levels in the indicated groups (n = 6 per group). (M) Mitochondrial membrane potential in the indicated groups (n = 6 per group). (N) Intracellular reactive oxygen species (ROS) levels measured by DCFH‐DA fluorescence after the indicated treatments (n = 6 per group). (O,P) Representative images of tumors and tumor weight in orthotopic pancreatic cancer models treated with control, shNCLX, EP, or shNCLX + EP (n = 5 per group). (Q) Kaplan–Meier survival curves of mice bearing orthotopic tumors under the same treatment conditions (n = 6 per group). (R) Based on Panc‐1 RNA‐seq data, comparison of NLRP3 and IL1B mRNA expression between WT and relatively EP‐resistant groups (n = 3 per group). (S) Western blot analysis of pyroptosis‐related proteins (N‐GSDMD, p‐p65, NLRP3, and cleaved caspase‐1) in Panc‐1 cells (Control, EP, siNCLX, and EP + siNCLX) and KPC‐A548 cells (Control, EP, shNCLX, and EP + shNCLX). (n = 3 per group). Data are presented as mean ± s.e.m. Statistical significance was assessed using the log‐rank (Mantel–Cox) test, two‐tailed Student's t test, or one‐way ANOVA. **p < 0.01; ***p < 0.001; ****p < 0.0001.

To define the molecular determinants underlying this altered mitochondrial Ca^2+^ homeostasis, we next focused on key regulators of mitochondrial Ca^2+^ transport. RNA sequencing (RNA‐seq) analysis revealed a pronounced upregulation of SLC8B1, which encodes the mitochondrial Ca^2+^ efflux exchanger NCLX, whereas genes associated with mitochondrial Ca^2+^ uptake showed no overt changes (Figure 2E). Consistently, NCLX mRNA and protein levels were significantly increased in relatively EP‐resistant Panc‐1 cells (Figure 2F,G). Immunohistochemical staining further confirmed elevated NCLX expression in tumor cells that survived EP treatment (Figure 2H). Moreover, based on an integrated analysis of the TCGA‐PAAD and GTEx datasets, NCLX expression was significantly higher in PDAC specimens than in normal pancreatic tissues (Figure S2D), implying that enhanced mitochondrial Ca^2+^ extrusion may represent an intrinsic feature of pancreatic cancer cells.

Given the robust induction of NCLX in EP‐resistant cells, we next assessed its functional contribution to EP sensitivity. Genetic knockdown of NCLX markedly reduced post‐EP survival (Figure 2I,J and Figure S2E–H). In contrast, NCLX overexpression significantly increased cell survival following EP treatment (Figure 2K and Figure S2I). Mechanistically, NCLX knockdown resulted in a pronounced elevation of mitochondrial Ca^2+^ following EP (Figure 2L), greater collapse of mitochondrial membrane potential (Figure 2M), and increased reactive oxygen species (ROS) production (Figure 2N), whereas NCLX overexpression substantially mitigated these effects (Figure 2L–N). In vivo, EP treatment of orthotopic tumors derived from NCLX‐deficient KPC A548 cells led to significantly reduced tumor volume and weight, together with a marked extension of mouse survival (Figure 2O–Q). Collectively, these findings support a model in which NCLX preserves mitochondrial Ca^2+^ homeostasis, limits EP‐triggered mitochondrial dysfunction, and thereby contributes to the enhanced post‐EP survival of pancreatic cancer cells.

Because prior studies suggest that mitochondrial Ca^2+^ overload can initiate inflammatory cell death programs, including pyroptosis, we performed an initial assessment of pyroptosis‐associated signaling in relatively EP‐resistant cells. Transcriptomic analysis showed that multiple pyroptosis‐related genes, including NLRP3 and IL1B, were expressed at significantly lower levels in EP‐resistant cells, indicating that survival after EP may be accompanied by suppression of the pyroptotic pathway (Figure 2R). Consistent with this notion, in both human‐ and murine‐derived pancreatic cancer cells, NCLX knockdown enhanced the expression of pyroptosis‐associated proteins under EP conditions. Functionally, the reduction in post‐EP survival caused by NCLX knockdown was partially reversed by a pyroptosis inhibitor (Figure 2S and Figure S3A–H). Furthermore, in vivo analysis showed that Nclx knockdown in KPC tumors reduced Ki67 expression and increased cle‐CASP1 levels following EP treatment (Figure S3I–K), providing additional evidence that EP combined with shNCLX promotes pyroptosis in pancreatic cancer cells.

Together, these findings suggest that NCLX‐dependent control of mitochondrial Ca^2+^ homeostasis may increase the resistance threshold to EP‐induced Ca^2+^ stress, thereby restraining pyroptosis and supporting the survival of a subset of pancreatic cancer cells after EP treatment. Nevertheless, whether pyroptosis represents a central mechanism responsible for the enhanced cytotoxicity induced by EP‐based Ca^2+^ stress requires further systematic investigation.

### 2.3 Development of a Mitochondrial Ca^2+^ Overload Strategy and Its Potentiated Cytotoxic Effect

We next reasoned that simultaneous blockade of mitochondrial Ca^2+^ extrusion and augmentation of Ca^2+^ burden immediately after EP could amplify mitochondrial Ca^2+^ stress and enhance tumor cell killing. Accordingly, we developed a therapeutic strategy designed to induce mitochondrial Ca^2+^ overload by combining EP with NCLX inhibition and exogenous Ca^2+^ delivery.

CGP37157 is a commonly used pharmacological inhibitor of NCLX activity [22, 23]. In vitro, dose–response analyses showed that either CGP37157 alone or extracellular Ca^2+^ alone exerted only minimal cytotoxic effects on tumor cells within commonly used concentration ranges, with apparent inhibitory effects observed only at relatively high concentrations in both Panc‐1 and KPC A548 cells (Figures S4A–D). We further evaluated the effects of Ca^2+^ and CGP37157 supplementation immediately after EP using cell viability heatmaps. These analyses included EP+Ca^2+^, EP+CGP37157, and EP+Ca2^+^+CGP37157 conditions across increasing concentrations of extracellular Ca^2+^ and CGP37157 (Figure S4E,F). Under EP conditions, Ca^2+^ supplementation alone induced only a modest reduction in tumor cell viability, whereas EP+CGP37157 produced a stronger inhibitory effect. Notably, the combined EP+Ca2^+^+CGP37157 treatment resulted in the most pronounced decrease in cell viability, indicating that simultaneous Ca^2+^ supplementation and NCLX inhibition markedly potentiated EP‐induced cytotoxicity. These results suggest that, in the context of EP, increasing Ca^2+^ influx alone is insufficient to maximize tumor cell killing, whereas concurrently restricting mitochondrial Ca^2+^ efflux while increasing Ca^2+^ load can substantially amplify mitochondrial Ca^2+^ overload and enhance cytotoxicity.

Given the poor aqueous solubility of CGP37157, we encapsulated it into mPEG‐PCL polymeric micelles to improve its bioavailability (Figure 3A), thereby providing a more stable formulation for combination treatment. The resulting micelles exhibited a predominant size, with an average diameter of 73.6 nm (Figure 3B), high drug encapsulation efficiency of 84.3% (Figure 3C), and a uniform ellipsoidal morphology (Figure 3D). In vitro release studies further demonstrated a stable sustained‐release profile under physiological conditions, with approximately 50% of CGP37157 released within 24 h at 37°C and pH 7.4 (Figure 3E), supporting its suitability for subsequent combination treatment.

**FIGURE 3:**
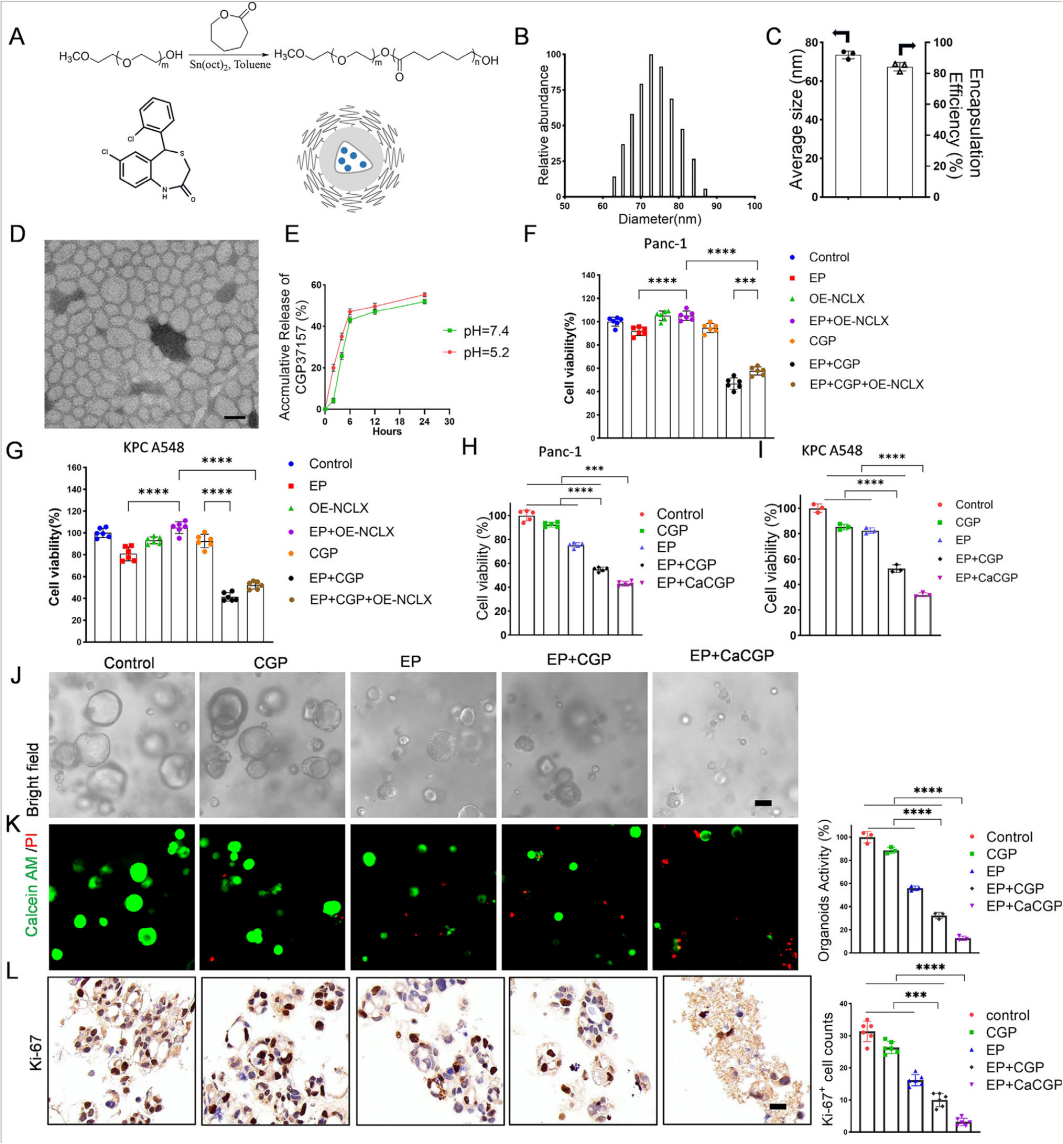
A mitochondrial Ca^2+^ overload strategy enhances EP‐induced cytotoxicity in pancreatic cancer cells and patient‐derived organoids. (A) Schematic illustration of the preparation of mPEG‐PCL@CGP37157 micelles. (B) Size distribution of mPEG‐PCL@CGP37157 micelles measured by dynamic light scattering (DLS). (C) Quantification of the hydrodynamic diameter and encapsulation efficiency of mPEG‐PCL@CGP37157 micelles (n = 3 per group). (D) Representative transmission electron microscopy (TEM) image of mPEG‐PCL@CGP37157 micelles. (E) In vitro cumulative release profile of CGP37157 from micelles at pH 7.4 and pH 5.2 (n = 3 per group). (F‐G) Cell viability of Panc‐1 and KPC A548 cells following the indicated treatments. (n = 6 per group). (H) Cell viability of Panc‐1 following the indicated treatments. EP refers to electroporation; CGP refers to mPEG‐PCL@CGP37157 micelles; Ca refers to exogenous Ca^2+^ supplementation; CaCGP refers to the combined local delivery of Ca^2+^ and CGP37157‐loaded micelles; and EP+CaCGP refers to electroporation followed by local co‐delivery of Ca^2+^ and CGP37157‐loaded micelles. (n = 5 per group). (I) Cell viability of KPC A548 cells following the indicated treatments. (n = 3 per group). (J) Representative bright‐field images of patient‐derived pancreatic cancer organoids after the indicated treatments (n = 3 per group). Scale bar = 50 µm. (K) Representative calcein AM/propidium iodide (PI) staining images (left) and quantification of organoid viability (right) after the indicated treatments. Live and dead cells are shown in green and red, respectively (n = 3 per group). Scale bar = 50 µm. (L) Representative Ki‐67 immunostaining images (left) and quantification of Ki‐67^+^ cells (right) in organoids after the indicated treatments (n = 6 per group). Scale bar = 50 µm. Data are presented as mean ± s.e.m. Statistical significance was assessed using a two‐tailed Student's *t*‐test or one‐way ANOVA. ***p < 0.001; ****p < 0.0001.

To determine whether CGP promotes pancreatic cancer cell death after EP primarily through inhibition of NCLX, we further examined its effect in NCLX‐overexpressing cells. As shown in Figure 3F,G, CGP markedly reduced the viability of OE‐NCLX pancreatic cancer cells following EP treatment, suggesting that pharmacological inhibition by CGP could overcome the protective effect conferred by NCLX overexpression. Because CGP has also been reported to potentially affect L‐type calcium channels, we next assessed whether L‐type calcium channel blockade alone could account for the enhanced cytotoxicity observed after EP. Compared with EP treatment alone, amlodipine combined with EP did not further reduce pancreatic cancer cell viability, whereas EP+CGP significantly suppressed cell viability (Figure S4G‐H). These results suggest that the cytotoxic effect of CGP after EP is unlikely to be mainly attributable to L‐type calcium channel inhibition. Instead, CGP appears to promote EP‐induced pancreatic cancer cell death predominantly through inhibition of NCLX‐mediated mitochondrial Ca^2+^ efflux.

Functionally, EP combined with mPEG‐PCL@CGP37157, equivalent to 10 µM free CGP37157, significantly reduced Panc‐1 cell survival, and the addition of 1 mM Ca^2+^ further potentiated cytotoxicity (Figure 3H). This combination treatment was termed EP+CaCGP, referring to electroporation followed by local co‐delivery of Ca^2+^ and CGP37157‐loaded micelles. A consistent inhibitory trend was observed in KPC A548 cells (Figure 3I). To assess the impact on long‐term proliferative capacity, we performed colony formation assays, which showed that EP+CaCGP markedly suppressed colony formation in both Panc‐1 (Figure S4I) and KPC A548 cells (Figure S4J), indicating that this combination not only reduces short‐term viability but also substantially impairs clonogenic growth.

To evaluate efficacy in a model that more closely recapitulates clinical disease, we further validated the strategy in patient‐derived organoids (PDOs). EP+CaCGP robustly inhibited overall organoid growth (Figure 3J), significantly decreased organoid viability (Figure 3K), and effectively suppressed cellular proliferation (Figure 3L). Collectively, these results demonstrate that the mitochondrial Ca^2+^ overload strategy achieved by combining EP with NCLX inhibition and Ca^2+^ delivery produces a pronounced combinatorial enhancement of cytotoxicity across multiple pancreatic cancer models, highlighting its translational potential for improving EP‐based therapeutic outcomes.

### 2.4 EP+CaCGP Triggers Mitochondrial Ca^2+^ Overload and Induces Mitochondrial Damage–Dependent Pyroptosis

Given that NCLX modulates cellular resistance to EP‐induced Ca^2+^ stress by regulating mitochondrial Ca^2+^ extrusion, we next systematically evaluated the effects of EP combined with NCLX inhibition and exogenous Ca^2+^ supplementation (EP+CaCGP) on mitochondrial homeostasis and cell death modality under conditions of intensified Ca^2+^ challenge. Rhod‐2 AM staining showed that EP+CaCGP markedly increased mitochondrial Ca^2+^ levels compared with control treatment (Figure 4A,B). Consistently, JC‐1 staining revealed a pronounced loss of mitochondrial membrane potential in the combination group (Figure 4C,D), accompanied by a significant elevation of intracellular reactive oxygen species (ROS) (Figure 4E,F). Transmission electron microscopy further confirmed severe mitochondrial ultrastructural damage in EP+CaCGP‐treated Panc‐1 cells, characterized by outer membrane rupture, disrupted or vanished cristae, and matrix vacuolization (Figure 4G). Together, these data indicate that EP+CaCGP induces profound mitochondrial dysfunction and structural collapse under EP and Ca^2+^‐intensive conditions.

**FIGURE 4:**
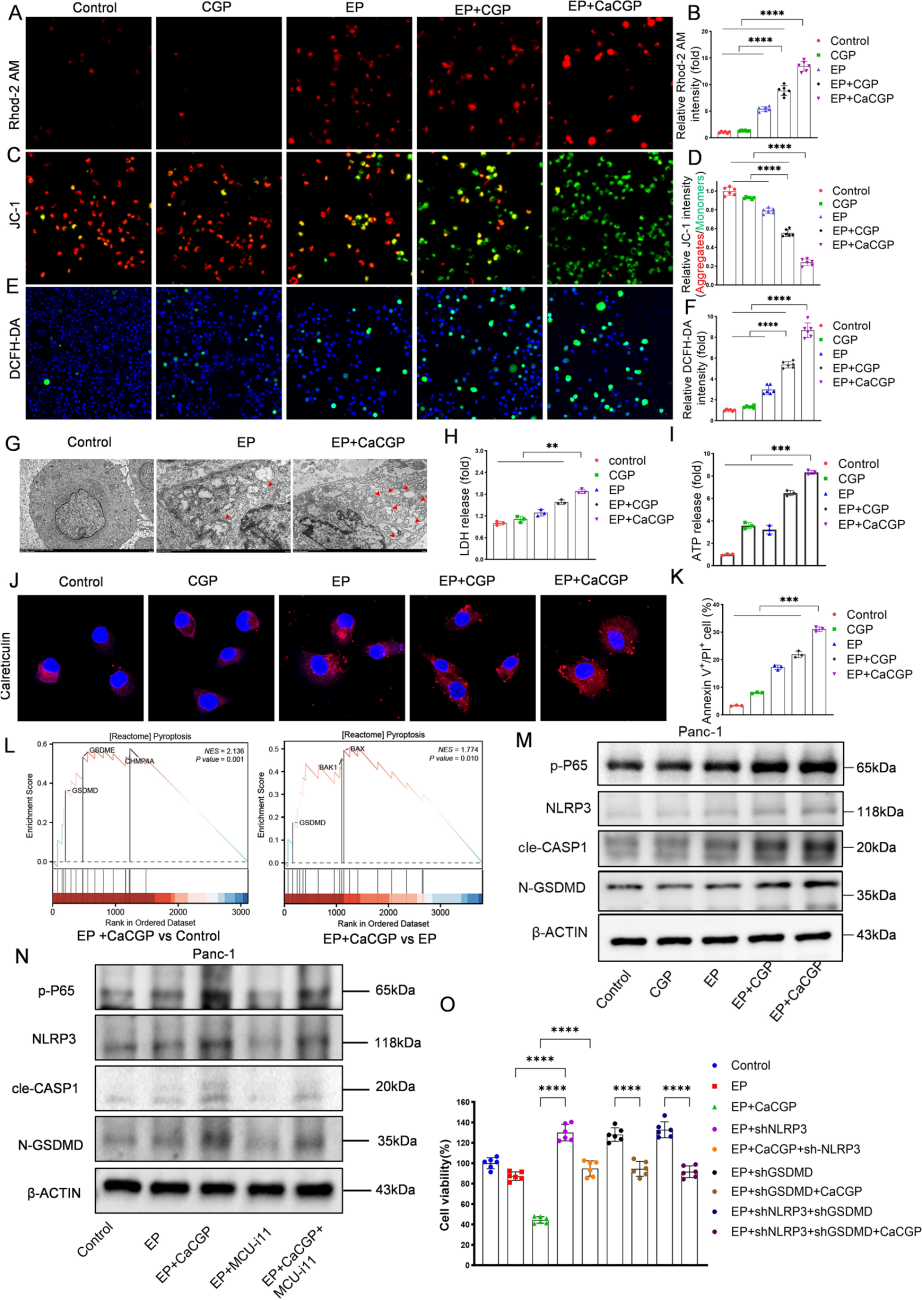
EP combined with NCLX inhibition and calcium induces mitochondrial damage, pyroptosis, and immunogenic cell death in pancreatic cancer cells. (A, B) Mitochondrial Ca^2+^ levels assessed by Rhod‐2 AM fluorescence in Panc‐1 cells after the indicated treatments (n = 6 per group). EP refers to electroporation; CGP refers to mPEG‐PCL@CGP37157 micelles; CaCGP refers to the combined local delivery of Ca^2+^ and CGP37157‐loaded micelles; and EP+CaCGP refers to electroporation followed by local co‐delivery of Ca^2+^ and CGP37157‐loaded micelles. (C, D) Mitochondrial membrane potential measured by JC‐1 staining in the corresponding groups (n = 6 per group). (E, F) Intracellular reactive oxygen species (ROS) levels determined by DCFH‐DA fluorescence after the indicated treatments (n = 6 per group). (G) Representative TEM images of Panc‐1 cells under the indicated conditions, showing severe mitochondrial ultrastructural disruption in the EP+CaCGP group (scale bar, 2.0 µm) (n = 6 per group). (H, I) Quantification of LDH release and extracellular ATP levels, indicating plasma membrane permeabilization and immunogenic cell death (n = 3 per group). (J) Cell‐surface exposure of calreticulin (CRT) after the indicated treatments (n = 3 per group). (K) Annexin V‐FITC/PI staining and quantification of Annexin V^+^/PI^+^ (membrane‐compromised) cells (n = 3 per group). (L) Gene set enrichment analysis (GSEA) showing enrichment of pyroptosis‐related pathways in EP+CaCGP‐treated cells (EP+CaCGP vs Control; EP+CaCGP vs EP). NES, Normalized enrichment score. (M) Western blot analysis of pyroptosis‐associated markers (N‐GSDMD, p‐P65, NLRP3, and cleaved CASP1) under the indicated conditions in Panc‐1 (n = 3 per group). (N) Western blot analysis of pyroptosis‐associated markers (N‐GSDMD, p‐P65, NLRP3, and cleaved CASP1) under the indicated conditions in Panc‐1 cells (n = 3 per group). (O) CCK8 analysis under the indicated conditions (Control, EP, EP+CaCGP, EP+shNLRP3, EP+shGSDMD, EP+CaCGP+ shNLRP3, EP+shNLRP3+shGSDMD, EP+CaCGP+ shGSDMD, EP+CaCGP+ shGSDMD+shNLRP3) in Panc‐1 (n = 6 per group). Data are presented as mean ± s.e.m. Statistical significance was assessed using a two‐tailed Student's *t*‐test or one‐way ANOVA. **p < 0.01; ***p < 0.001; ****p < 0.0001.

In agreement with a lytic cell death process, EP+CaCGP significantly increased lactate dehydrogenase (LDH) release (Figure 4H) and ATP efflux (Figure 4I), suggesting cell death accompanied by the release of damage‐associated molecular patterns (DAMPs). In parallel, immunofluorescence staining demonstrated evident calreticulin translocation to the cell surface following EP+CaCGP treatment (Figure 4J), further supporting the emergence of immunogenic features. Annexin V‐FITC/PI double staining additionally showed a marked increase in the double‐positive population in the EP+CaCGP group (Figure 4K), consistent with substantially enhanced membrane permeabilization during pyroptosis. Similar findings were observed in KPC A548 cells (Figures S5A–L).

At the mechanistic level, gene set enrichment analysis (GSEA) revealed significant enrichment of pyroptosis‐associated pathways in EP+CaCGP‐treated Panc‐1 cells (Figure 4L). Protein validation further demonstrated robust induction of multiple pyroptosis markers, including cleaved GSDMD (N‐GSDMD), phosphorylated p65 (p‐P65), NLRP3, and cleaved caspase‐1 (cleaved‐CASP1) (Figure 4M and Figure S6A–C), providing direct molecular evidence for pyroptosis activation in both Panc‐1 and KPC cells.

To further clarify whether mitochondrial Ca^2+^ overload functions upstream of pyroptotic signaling, we inhibited mitochondrial Ca^2+^ uptake using MCU‐i11. MCU‐i11 treatment attenuated EP+CaCGP‐induced tumor cell death and reduced the induction of pyroptosis‐associated proteins, including N‐GSDMD and cleaved‐CASP1 (Figure 4N and Figure S7A–C). These results suggest that mitochondrial Ca^2+^ uptake and subsequent mitochondrial Ca^2+^ overload are required for efficient activation of CASP1/GSDMD‐dependent pyroptotic signaling after EP+CaCGP treatment.

We next examined whether the canonical pyroptotic machinery was functionally involved in this process. NLRP3 knockdown in both Panc‐1 and KPC A548 cells significantly rescued cells from EP+CaCGP‐induced death, indicating that NLRP3 inflammasome activation contributes to this cell death process (Figure 4O and Figure S7D). In parallel, GSDMD knockdown also attenuated EP+CaCGP‐induced cell death in both cell lines, further supporting GSDMD‐mediated pyroptosis as an important execution mechanism of EP+CaCGP‐induced cytotoxicity (Figure 4O and Figure S7D).

Collectively, these results support a mechanistic model in which EP+CaCGP induces mitochondrial Ca^2+^ overload and mitochondrial damage, thereby promoting activation of the NLRP3/CASP1/GSDMD pyroptotic pathway and contributing to pancreatic cancer cell death.

### 2.5 EP+CaCGP Markedly Suppresses PDAC Tumor Progression In Vivo and Prolongs Survival

To evaluate the in vivo anti‐tumor efficacy of electroporation combined with NCLX inhibition and Ca^2+^ supplementation (EP+CaCGP), we first tested this regimen in a KPC A548 subcutaneous tumor model (Figure 5A). CaCGP alone exerted only limited effects on tumor growth, whereas EP alone transiently delayed tumor progression but was followed by tumor relapse. In contrast, EP+CaCGP produced substantially stronger tumor control (Figure 5B). At the two‐week endpoint, the combination treatment exhibited markedly reduced tumor volume and tumor weight (Figure 5C,D), accompanied by a significant improvement in overall survival (Figure 5E).

**FIGURE 5:**
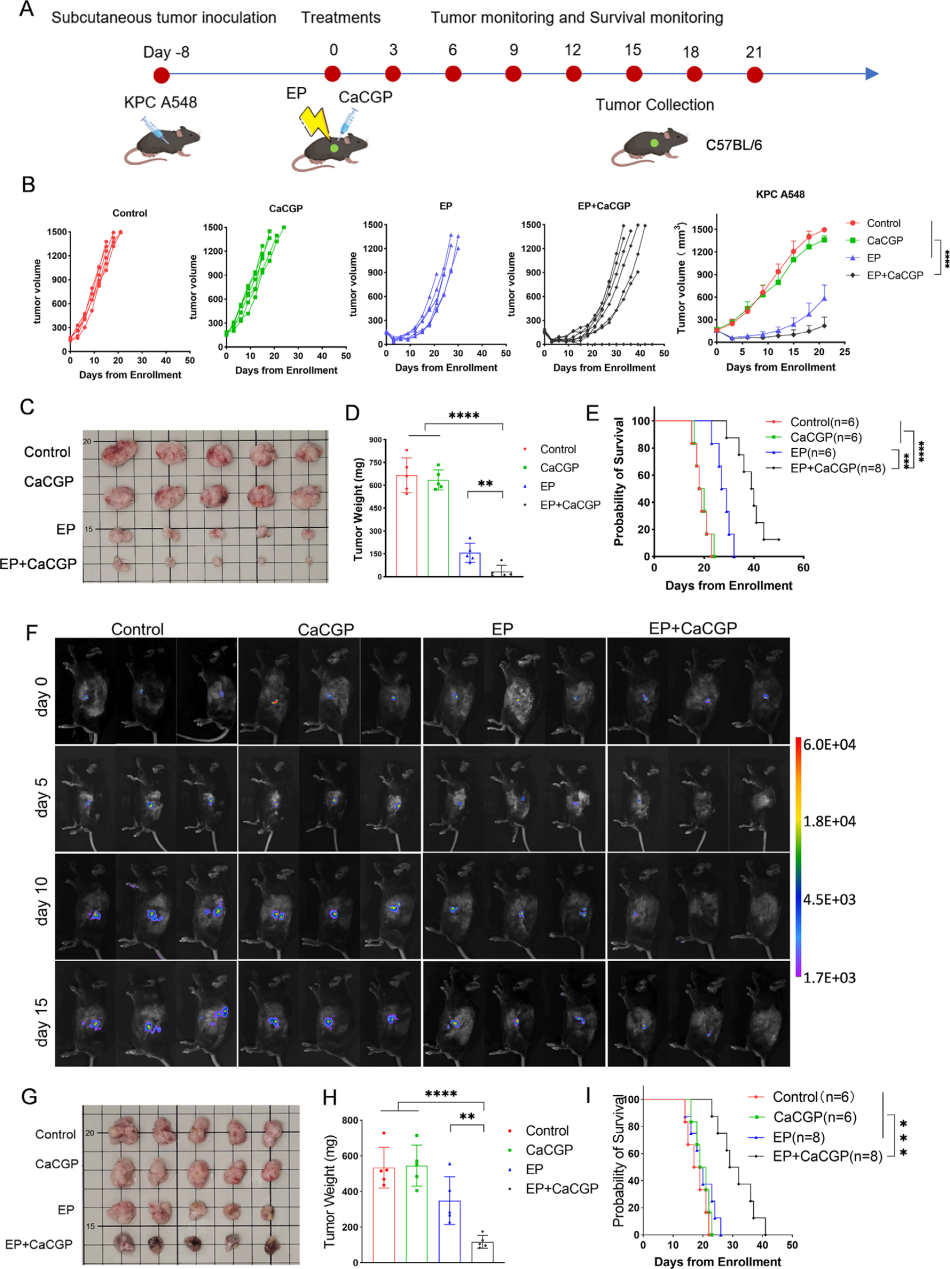
EP combined with NCLX inhibition and calcium suppresses tumor growth and prolongs survival in mouse pancreatic cancer models. (A) Schematic illustration of the treatment regimen in mice bearing subcutaneous KPC A548 tumors. (B) Individual (left) and mean (right) tumor volume growth curves for the indicated treatment groups (Control n = 6; CaCGP n = 6; EP n = 6; EP+CaCGP n = 8). EP refers to electroporation; EP+CaCGP refers to electroporation followed by local co‐delivery of Ca^2+^ and CGP37157‐loaded micelles. (C, D) Representative images and quantification of tumor weights measured two weeks after treatment (n = 5 per group). (E) Kaplan–Meier survival curves of subcutaneous tumor‐bearing mice receiving the indicated treatments (Control n = 6; CaCGP n = 6; EP n = 6; EP+CaCGP n = 8). (F) Representative in vivo bioluminescence imaging of orthotopic pancreatic tumors at the indicated time points after treatment (n = 3 per group). (G, H) Representative images and quantification of orthotopic tumor weights measured two weeks after treatment (n = 5 per group). (I) Kaplan–Meier survival curves of mice bearing orthotopic pancreatic tumors under the indicated treatment conditions (Control n = 6; CaCGP n = 6; EP n = 8; EP+CaCGP n = 8). Data are presented as mean ± s.e.m. Statistical significance was assessed using the log‐rank (Mantel–Cox) test, two‐tailed Student's *t* test, or one‐way ANOVA. **p < 0.01; ****p < 0.0001.

We next examined the efficacy and reproducibility of this combinatorial strategy in an orthotopic pancreatic cancer model. In vivo imaging revealed that EP+CaCGP significantly restrained orthotopic tumor growth kinetics (Figure 5F). Endpoint analyses further confirmed that tumor volume and tumor weight were both significantly reduced in the combination group (Figure 5G,H), together with a pronounced survival benefit (Figure 5I). IHC analysis further showed that EP+CaCGP inhibits pancreatic cancer cell proliferation and promotes pyroptosis in tumor tissues, as evidenced by reduced Ki‐67 staining and increased pyroptosis‐related marker expression (Figure S8A,B).

Collectively, EP+CaCGP achieved consistent and robust therapeutic efficacy across both subcutaneous and orthotopic pancreatic cancer models. Compared with monotherapies, this combination regimen significantly reduced tumor burden and improved survival outcomes, highlighting its potential for further translational development.

### 2.6 EP+CaCGP Induces Immunogenic Cell Death via Mitochondrial Ca^2+^ Overload and Drives Antitumor Immunity

#### 2.6.1 EP+CaCGP‐Induced Tumor Cell Death Promotes APC Activation and T‐Cell Responses In Vitro

Given that pyroptosis represents a prototypical form of immunogenic cell death (ICD), we investigated whether tumor cells subjected to EP+CaCGP acquire immunostimulatory capacity in vitro. Using a co‐culture system incorporating dendritic cells (DCs), macrophages, and T cells (Figure 6A–F), we found that EP+CaCGP‐treated tumor cells markedly promoted DC maturation, as indicated by the upregulation of maturation‐associated markers (Figure 6G,H), and induced macrophage polarization toward an M1‐like phenotype (Figure 6I,J). In parallel, T‐cell activation was significantly enhanced in the same system (Figure 6K,L). Collectively, these results demonstrate that EP+CaCGP‐induced tumor cell death is immunostimulatory and can initiate key cellular processes required for antitumor immune responses in vitro.

**FIGURE 6:**
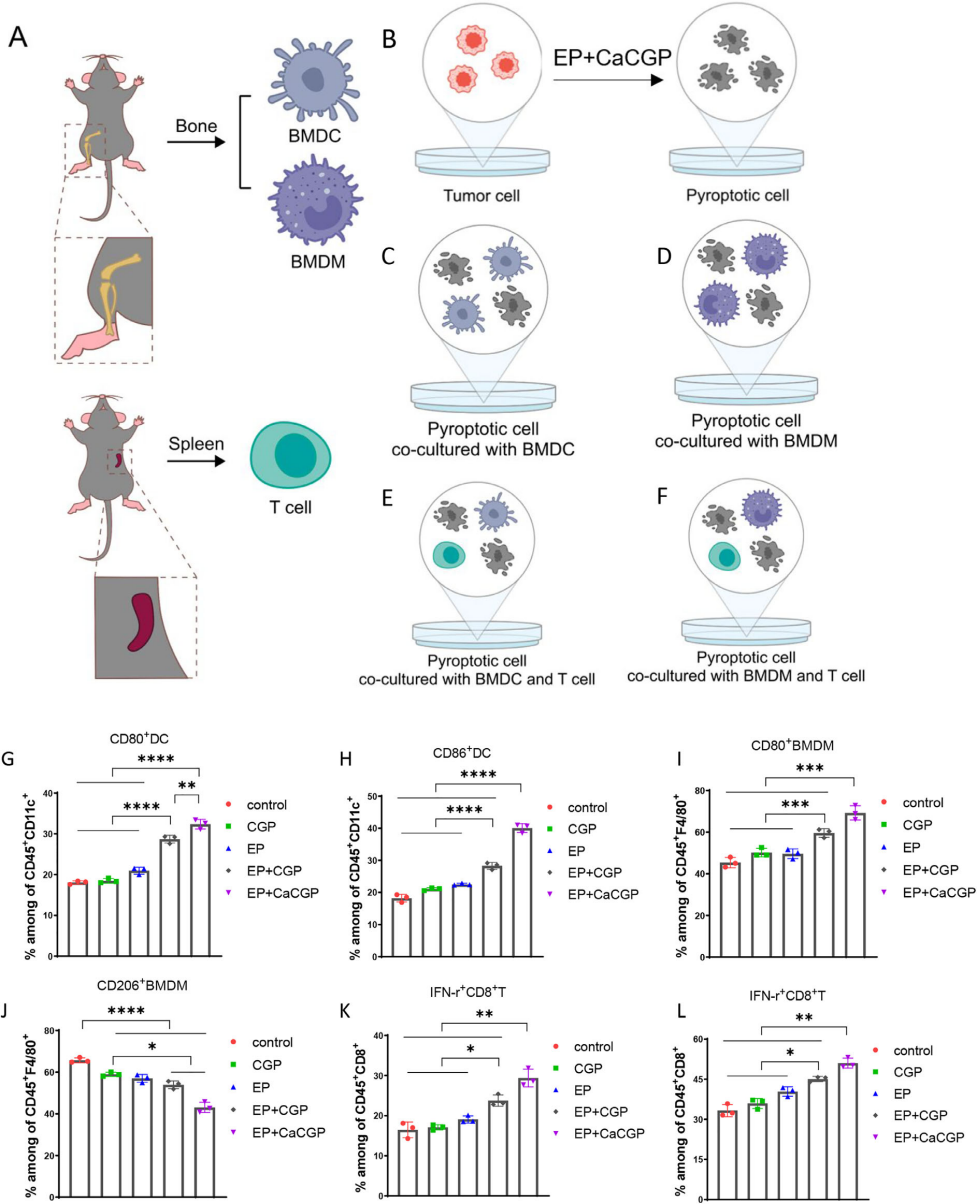
EP+CaCGP‐treated tumor cells stimulate antigen‐presenting cell activation and CD8^+^ T‐cell responses in vitro. (A) Schematic illustrating the isolation of bone marrow–derived dendritic cells (BMDCs) and bone marrow–derived macrophages (BMDMs) from mouse bone marrow, and T cells from the spleen. (B–F) Schematic of the co‐culture systems. Tumor cells were subjected to the indicated treatments to generate pyroptotic tumor cells (B), which were then co‐cultured with BMDCs (C), BMDMs (D), BMDCs plus T cells (E), or BMDMs plus T cells (F). EP refers to electroporation; CaCGP refers to the combined local delivery of Ca^2+^ and CGP37157‐loaded micelles; and EP+CaCGP refers to electroporation followed by local co‐delivery of Ca^2+^ and CGP37157‐loaded micelles. (G, H) Flow cytometry quantification of BMDC maturation, shown as CD80^+^ or CD86^+^ cells among CD45^+^CD11c^+^ cells after co‐culture with treated tumor cells (n = 3 per group). (I, J) Flow cytometry quantification of BMDM polarization, shown as CD80^+^ (M1‐like) or CD206^+^ (M2‐like) cells among CD45^+^F4/80^+^ cells after co‐culture with treated tumor cells (n = 3 per group). (K, L) Flow cytometry quantification of CD8^+^ T‐cell activation, shown as IFN‐γ^+^ cells among CD45^+^CD8^+^ T cells in the BMDC–T cell co‐culture system (E) and the BMDM–T cell co‐culture system (F) (n = 3 per group). Data are presented as mean ± s.e.m. Statistical significance was assessed using one‐way ANOVA. *p < 0.05; **p < 0.01; ***p < 0.001; ****p < 0.0001.

#### 2.6.2 NCLX Inhibition Synergizes With EP to Enhance Local Effector Immunity Within Tumors

To validate the immunomodulatory role of NCLX inhibition in vivo while minimizing potential confounding effects from drug carriers, genetic NCLX suppression was combined with EP treatment. Multicolor immunofluorescence analyses showed that, compared with EP alone or control treatment, EP combined with NCLX inhibition significantly strengthened local effector immunity within tumors (Figure S9A,B). This was characterized by increased total CD8^+^ T cells and an expanded IFN‐γ^+^ CD8^+^ subset, together with elevated DC abundance and a higher proportion of CD86^+^ mature DCs. Concomitantly, the intratumoral myeloid compartment shifted toward an antitumor phenotype, as evidenced by reduced M2‐like macrophages and increased M1‐like macrophage features. These findings suggest that NCLX inhibition not only alters tumor cell fate by restricting mitochondrial Ca^2+^ extrusion but also reshapes the local immune microenvironment toward an effector‐oriented state, providing an in vivo rationale for the subsequent development of the CGP37157‐based EP+CaCGP regimen.

#### 2.6.3 EP+CaCGP Remodels the Tumor Microenvironment and Augments Intratumoral Effector Immunity

Transcriptomic profiling at day 4 after treatment revealed extensive immune remodeling in the EP+CaCGP group (Figure 7A,B), characterized by increased infiltration of T cells and macrophages. Within the T‐cell compartment, effector and cytotoxic CD8^+^ T‐cell populations were enriched, whereas exhausted CD8^+^ T‐cell subsets were reduced (Figures 7C–D). In parallel, macrophages shifted toward an M1‐like inflammatory phenotype (Figure 7E,F), and the proportion of CD86^+^ dendritic cells increased (Figure 7G), consistent with enhanced antigen‐presenting activity.

**FIGURE 7:**
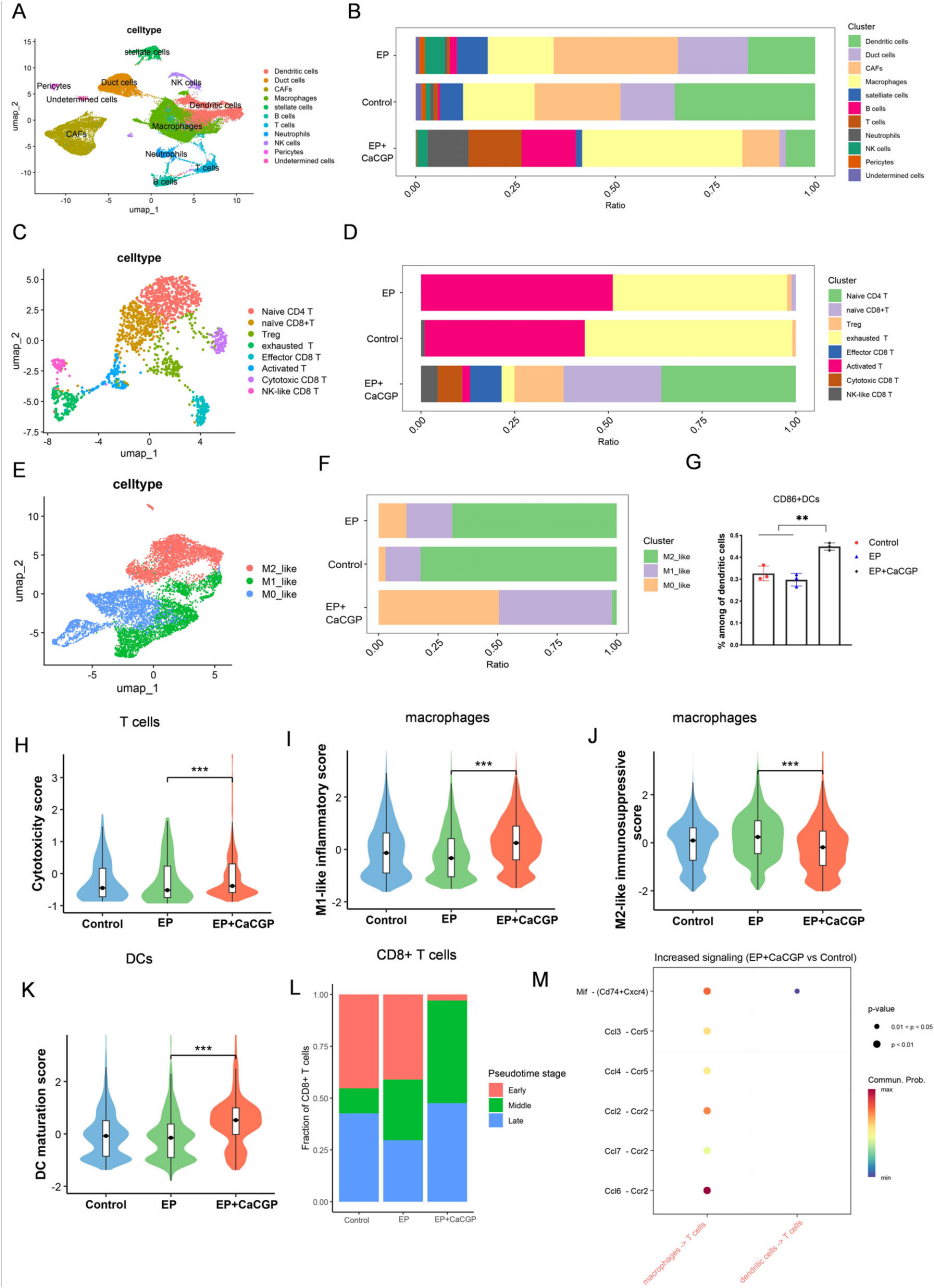
EP+CaCGP remodels the tumor microenvironment. (A, B) Single‐cell RNA sequencing (scRNA‐seq) overview of tumors at day 4 post‐treatment, showing global tumor microenvironment (TME) composition and treatment‐associated remodeling (n = 3 per group; visualized as indicated). EP refers to electroporation; CGP refers to mPEG‐PCL@CGP37157 micelles; Ca refers to exogenous Ca^2+^ supplementation; CaCGP refers to the combined local delivery of Ca^2+^ and CGP37157‐loaded micelles; and EP+CaCGP refers to electroporation followed by local co‐delivery of Ca^2+^ and CGP37157‐loaded micelles. (C, D) scRNA‐seq analysis of the T‐cell compartment, showing increased effector/cytotoxic CD8^+^ T‐cell subsets and reduced exhausted T‐cell subsets in the EP+CaCGP group (n = 3 per group). (E, F) scRNA‐seq analysis of macrophage populations, showing a shift toward an M1‐like transcriptional state following EP+CaCGP treatment (n = 3 per group). (G) scRNA‐seq quantification of CD86^+^ dendritic cells, indicating increased DC maturation in the EP+CaCGP group (n = 3 per group). (H) Module‐score analysis of T‐cell functional states, showing cytotoxicity scores in the indicated treatment groups (n = 3 per group). (I‐J) Module‐score analysis of macrophage functional states, showing M1‐like inflammatory and M2‐like immunosuppressive scores in the indicated treatment groups (n = 3 per group). (K) Module‐score analysis of dendritic‐cell maturation states, showing DC maturation scores in the indicated treatment groups (n = 3 per group). (L) Pseudotime trajectory analysis of CD8^+^ T‐cell subsets after the indicated treatments, showing enrichment of CD8^+^ T cells from the EP+CaCGP group in middle‐to‐late pseudotime states (n = 3 per group). (M) CellChat‐based ligand–receptor interaction analysis between myeloid cells and T cells, highlighting enhanced CCL6–CCR2 and MIF–CD74/CXCR4 interactions between macrophages or dendritic cells and T cells in the EP+CaCGP group (n = 3 per group). Data are presented as mean ± s.e.m. Statistical significance was assessed using one‐way ANOVA. **p < 0.01; ***p < 0.001.

Functional‐state scoring using AddModuleScore further indicated that EP+CaCGP reinforced antitumor immune programs across major immune subsets. Compared with EP alone, EP+CaCGP increased cytotoxicity‐associated scores in T cells (Figure 7H). In macrophages, EP+CaCGP enhanced the M1‐like inflammatory score while reducing the M2‐like immunosuppressive score (Figure 7I,J). In dendritic cells, the DC maturation score was also elevated following EP+CaCGP treatment (Figure 7K). These findings indicate that EP+CaCGP not only reshapes immune‐cell composition within the TME but also promotes antitumor functional activation of T cells, macrophages, and dendritic cells.

Pseudotime trajectory analysis revealed a treatment‐associated shift in CD8^+^ T‐cell state transitions. CD8^+^ T cells from the EP+CaCGP group were preferentially enriched in the middle‐to‐late stages of the trajectory, whereas those from the EP group were relatively enriched in earlier states (Figure 7L). Naive CD8^+^ T cells were mainly distributed at the early stage of pseudotime, activated and effector CD8^+^ T cells accumulated in the intermediate stage, and cytotoxic CD8^+^ T cells tended to localize to the late stage (Figure S10A). These findings suggest that EP+CaCGP promotes CD8^+^ T‐cell progression from naive/early states toward activated, effector, and cytotoxic states.

CellChat analysis further showed that EP+CaCGP reshaped intercellular communication within the TME. Global cell–cell communication analysis revealed increased interaction numbers between macrophages and T cells, as well as between dendritic cells and T cells, after EP+CaCGP treatment (Figure S10B,C). Focused ligand–receptor analysis identified enhanced CCL6–CCR2 and MIF–CD74/CXCR4 interactions between macrophages or dendritic cells and T cells in the EP+CaCGP group (Figure 7M). These results suggest that EP+CaCGP may strengthen myeloid–T cell communication networks that support T‐cell‐mediated antitumor immunity.

Collectively, the Single‐cell RNA sequencing (scRNA‐seq) data indicate that EP+CaCGP converts the TME from an immunosuppressive state toward an immune‐activated state by promoting CD8^+^ T‐cell activation and cytotoxic differentiation, enhancing antitumor macrophage and dendritic‐cell functional states, and reinforcing immune‐regulatory communication between myeloid cells and T cells.

To corroborate these transcriptomic findings, we performed orthogonal validation using flow cytometry and multicolor immunofluorescence. Flow cytometric analyses showed increased infiltration of total T cells, CD8^+^ T cells, and IFN‐γ^+^ CD8^+^ T cells in EP+CaCGP‐treated tumors, accompanied by a marked reduction in regulatory T cells (Tregs) (Figure 8A–D). Consistent with enhanced myeloid immune activation, EP+CaCGP also increased the proportion of CD86^+^ dendritic cells and significantly elevated the macrophage M1/M2 ratio (Figure 8E–H). The gating strategy for flow cytometry is shown in Figure S11. Multicolor immunofluorescence imaging further confirmed the in situ enrichment of CD8^+^ T cells, mature dendritic cells, and M1‐like macrophages within EP+CaCGP‐treated tumors (Figure 8I–O). Collectively, these integrated transcriptomic, cytometric, and tissue‐imaging data demonstrate that EP+CaCGP robustly activates local antitumor immunity in vivo.

**FIGURE 8:**
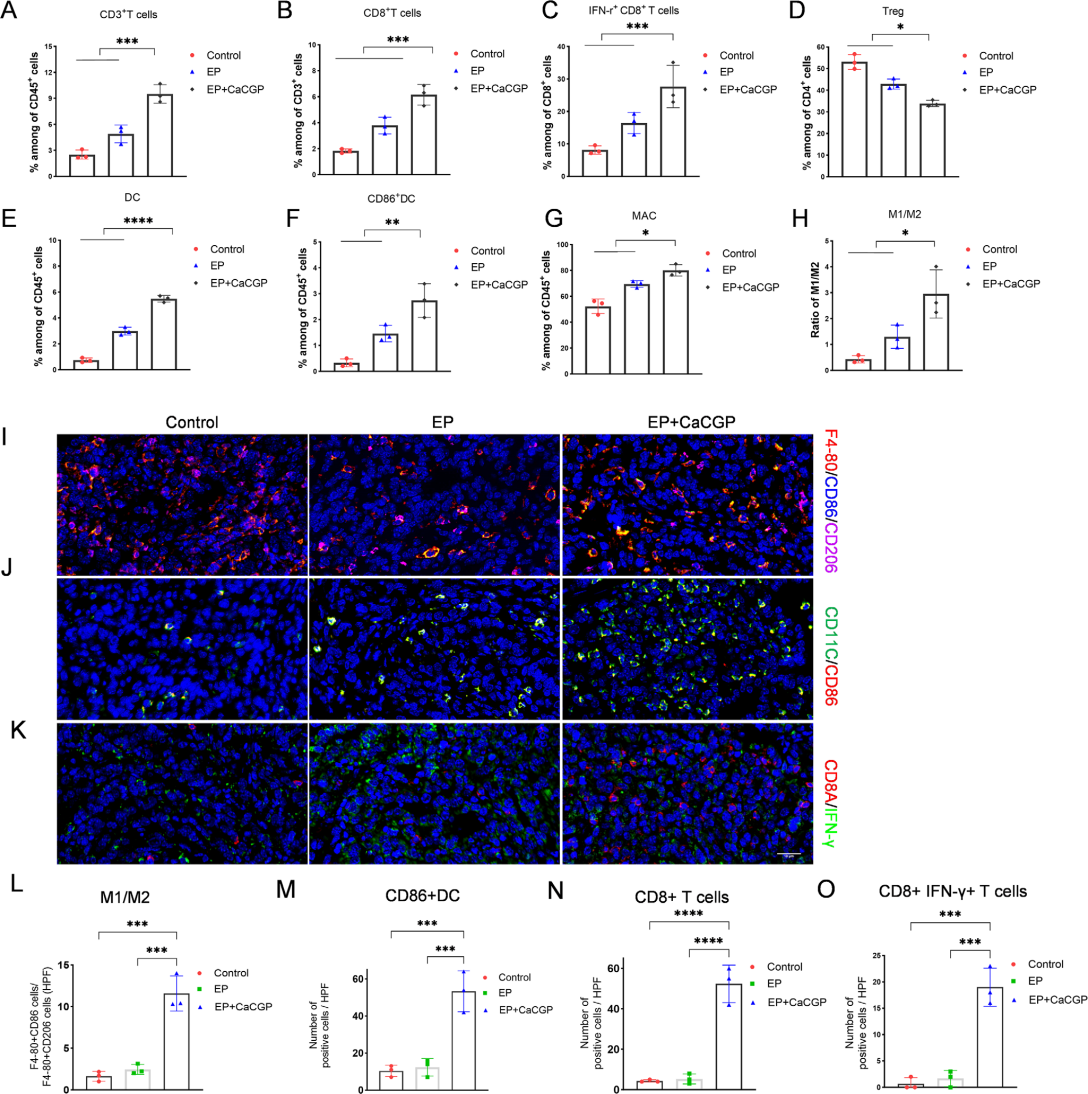
Orthogonal validation confirms that EP+CaCGP activates intratumoral effector immunity in vivo. (A–D) Flow cytometry quantification of tumor‐infiltrating T‐cell populations in the indicated groups, including total CD3^+^ T cells, CD8^+^ T cells, IFN‐γ^+^ CD8^+^ T cells, and regulatory T cells (Tregs) (n = 3 per group). EP refers to electroporation; CaCGP refers to the combined local delivery of Ca^2+^ and CGP37157‐loaded micelles; and EP+CaCGP refers to electroporation followed by local co‐delivery of Ca^2+^ and CGP37157‐loaded micelles. (E–H) Flow cytometry quantification of myeloid immune populations in tumors, including total dendritic cells (DCs), CD86^+^ mature DCs, macrophages, and the M1/M2 macrophage ratio in the indicated groups (n = 3 per group). (I–K) Representative multicolor immunofluorescence images of tumor sections from each group, showing macrophage markers and polarization (F4/80, CD86, CD206), DC markers (CD11c, CD86), T‐cell infiltration (CD8), and effector activation (CD8, IFN‐γ) (markers as indicated) (scale bar, 10 µm). (L‐O) Quantification of multicolor immunofluorescence staining in (I–K), including the M1/M2 macrophage ratio, CD86^+^ DCs, CD8^+^ T cells, and CD8^+^IFN‐γ^+^ T cells per high‐power field (HPF) (n = 3 per group). Data are presented as mean ± s.e.m. Statistical significance was assessed using a two‐tailed Student's *t*‐test or one‐way ANOVA. *p < 0.05; **p < 0.01; ***p < 0.001; ****p < 0.0001.

#### 2.6.4 EP+CaCGP Elicits Systemic Immune Activation and Suppresses Liver Metastasis

To define the functional contribution of adaptive immune responses to therapeutic efficacy, we first evaluated EP+CaCGP in nude mice lacking functional T cells. In this T‐cell‐deficient setting, the antitumor efficacy of EP+CaCGP was markedly attenuated (Figure 9A–C), and the survival benefit observed in immunocompetent mice was abolished. Consistently, in the nude mouse orthotopic tumor model, EP+CaCGP only partially increased pyroptosis and failed to markedly inhibit tumor cell proliferation, as reflected by cleaved CASP1 and Ki‐67 staining (Figure S12A,B). These results indicate that although EP+CaCGP can directly induce tumor‐cell stress and pyroptotic signaling, its full therapeutic efficacy in vivo requires functional T‐cell immunity.

**FIGURE 9:**
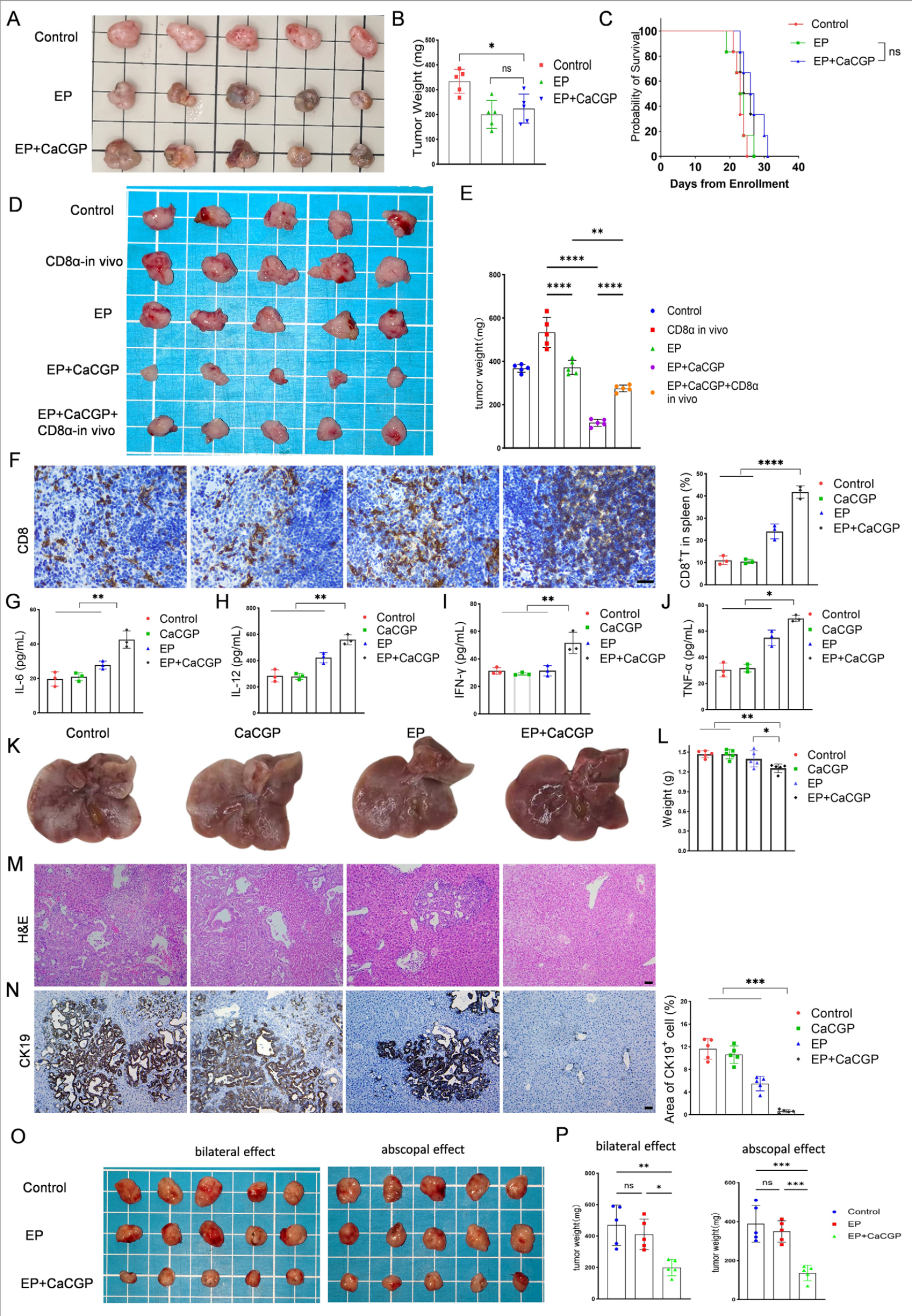
EP+CaCGP requires T‐cell immunity, induces systemic immune activation, and suppresses liver metastasis. (A) Representative images of tumors from T‐cell–deficient nude mice treated with the indicated regimens (n = 5 per group). EP refers to electroporation; CaCGP refers to the combined local delivery of Ca^2+^ and CGP37157‐loaded micelles; and EP+CaCGP refers to electroporation followed by local co‐delivery of Ca^2+^ and CGP37157‐loaded micelles. (B) Tumor weight in nude mice after the indicated treatments (n = 5 per group). (C) Kaplan–Meier survival curves of nude mice bearing tumors under the indicated treatment conditions (n = 6 per group). (D‐E) Representative tumor images (D) and quantification of tumor weight (E) from mice treated with Control, anti‐CD8α, EP, EP+CaCGP, or EP+CaCGP + anti‐CD8α, showing that CD8^+^ T‐cell depletion attenuates the tumor‐suppressive effect of EP+CaCGP (n = 5 per group). (F) Representative CD8 immunohistochemical staining of spleen sections (left) and quantification of splenic CD8^+^ cells (right) after the indicated treatments (n = 3 per group; scale bar = 50 µm). (G–J) Serum cytokine levels (IL‐6, IL‐12, IFN‐γ, and TNF‐α) measured after the indicated treatments (n = 3 per group). (K‐L) Representative images of livers after the indicated treatments; Liver weight in the liver metastasis model (n = 5 per group). (M) Representative H&E staining of liver sections showing metastatic lesions in the indicated groups (scale bar as indicated) (n = 5 per group). (N) Representative CK19 immunohistochemical staining of liver sections (left) and quantification of CK19^+^ metastatic lesion area (right) (n = 5 per group; scale bar = 50 µm). (O) Representative endpoint images in the bilateral subcutaneous tumor model and Orthotopic pancreatic tumor–distal subcutaneous tumor model assessing systemic antitumor effects under the indicated groups (n = 5 per group). (P) Quantification of untreated contralateral tumor burden in the bilateral subcutaneous tumor model and orthotopic pancreatic tumor–distal subcutaneous tumor model assessing systemic antitumor effects under the indicated groups (n = 5 per group). Data are presented as mean ± s.e.m. Statistical significance was assessed using the log‐rank (Mantel–Cox) test, two‐tailed Student's *t* test, or one‐way ANOVA. *p < 0.05; **p < 0.01; ***p < 0.001; ****p < 0.0001; ns, not significant.

To further identify the effector T‐cell subset responsible for this immune‐dependent antitumor activity, we performed in vivo CD8^+^ T‐cell depletion using an anti‐CD8α antibody. EP+CaCGP markedly reduced tumor burden and prolonged survival in control mice; however, these therapeutic effects were largely abolished following CD8^+^ T‐cell depletion (Figure 9D,E and Figure S12C). These findings demonstrate that CD8^+^ T cells are critical mediators of EP+CaCGP‐induced antitumor efficacy. In line with this immune‐dependent mechanism, systemic immune profiling showed that EP+CaCGP increased the proportion of splenic CD8^+^ T cells (Figure 9F) and significantly elevated serum levels of IL‐6, IL‐12, IFN‐γ, and TNF‐α (Figure 9G–J), indicating robust systemic immune activation.

The systemic antitumor activity of EP+CaCGP was further evaluated in a KPC A548 liver metastasis model. EP+CaCGP treatment conferred pronounced protection against metastatic progression, as evidenced by the lowest liver weight among the treatment groups and a significant reduction in metastatic lesion area confirmed by H&E staining and CK19 immunohistochemistry (Figure 9K–N). These results suggest that EP+CaCGP not only remodels local antitumor immunity but also mobilizes systemic immune responses capable of restraining distant metastatic outgrowth.

To determine whether this systemic immune activation could control untreated distal tumors, we established a bilateral subcutaneous tumor model in which only one tumor received local treatment. Local EP+CaCGP treatment of the primary tumor significantly suppressed the growth of the untreated contralateral tumor compared with the control and EP groups (Figure 9O–P), indicating the induction of an abscopal antitumor effect. This distal tumor‐control effect was further validated in an orthotopic pancreatic tumor model combined with an untreated subcutaneous tumor. In this model, local EP+CaCGP treatment of the orthotopic pancreatic tumor markedly inhibited the growth of the distant untreated subcutaneous tumor (Figure 9O,P and Figure S12D). Together, these results demonstrate that EP+CaCGP induces CD8^+^ T‐cell‐dependent systemic antitumor immunity, thereby enabling both local tumor control and suppression of distant metastatic or untreated tumors.

### 2.7 Pyroptosis is Required for EP+CaCGP–Induced Antitumor Immunity and Therapeutic Efficacy In Vivo

The enrichment of pyroptosis markers, including cleaved Caspase‐1 and N‐GSDMD, after EP+CaCGP treatment (Figures S13A–B) suggested that pyroptotic cell death may contribute to the therapeutic response. Given the robust immune activation induced by EP+CaCGP, we further examined whether pyroptosis functions as a mechanistic link between mitochondrial Ca^2+^ overload and antitumor immunity.

Genetic disruption of CASP1‐mediated pyroptotic signaling attenuated the therapeutic efficacy of EP+CaCGP. In mouse tumor models established with CASP1‐knockdown KPC A548 cells, EP+CaCGP showed a weakened tumor‐suppressive effect compared with EP+CaCGP‐treated control tumors, as evidenced by increased tumor burden after CASP1 knockdown (Figure 10A,B). Consistently, CASP1 knockdown also reduced the survival benefit conferred by EP+CaCGP (Figure S13C). These findings provide genetic evidence that CASP1‐mediated pyroptosis contributes to the in vivo antitumor activity of EP+CaCGP.

**FIGURE 10:**
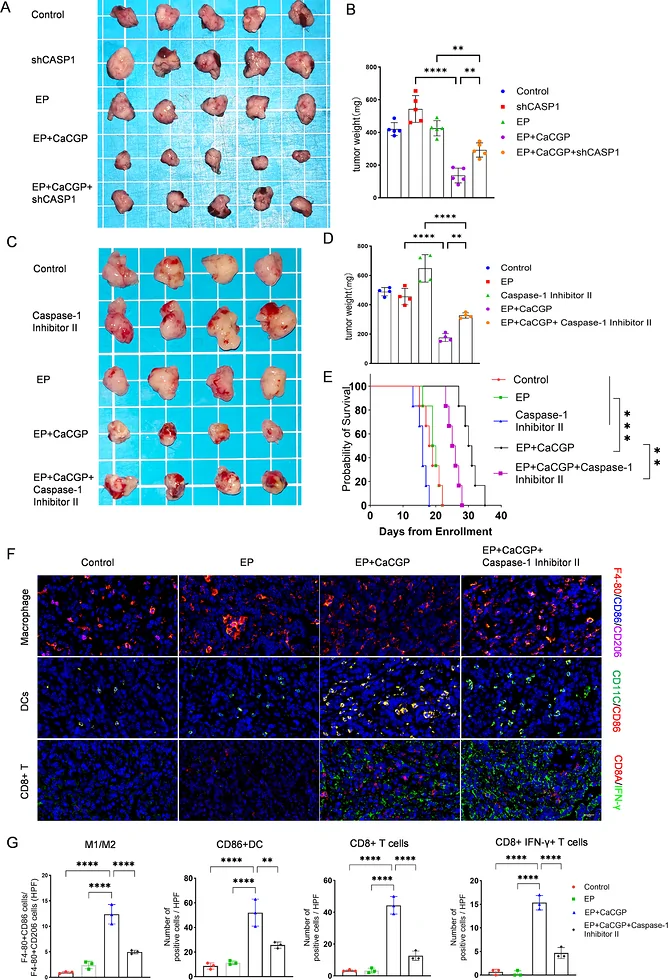
Pyroptosis is required for EP+CaCGP–induced antitumor immunity and therapeutic efficacy in vivo. (A, B) Representative tumor images (A) and quantification of tumor weight (B) from mice bearing control or CASP1‐knockdown KPC A548 tumors treated with EP+CaCGP, showing that CASP1 knockdown partially reverses the tumor‐suppressive effect of EP+CaCGP (n = 5 per group). EP refers to electroporation; CaCGP refers to the combined local delivery of Ca^2+^ and CGP37157‐loaded micelles; and EP+CaCGP refers to electroporation followed by local co‐delivery of Ca^2+^ and CGP37157‐loaded micelles. (C) Representative tumor images from the orthotopic PDAC model treated with Control, EP, Caspase‐1 Inhibitor II, EP+CaCGP, or EP+CaCGP + Caspase‐1 Inhibitor II (n = 4 per group). (D) Tumor weight in the orthotopic PDAC model after the indicated treatments (n = 4 per group). (E) Kaplan–Meier survival curves of orthotopic PDAC‐bearing mice receiving the indicated regimens (n = 6 per group). (F) Representative multicolor immunofluorescence images showing intratumoral macrophage polarization (F4/80, CD86, CD206), dendritic cell maturation (CD11c, CD86), and CD8^+^ T‐cell infiltration/activation (CD8A, IFN‐γ) under the indicated conditions (scale bar 10 um) (n = 3 per group). (G) Quantification of the M1/M2 macrophage ratio, CD86^+^ DCs, CD8^+^ T cells, and CD8^+^ IFN‐γ^+^ T cells per high‐power field (HPF) from multicolor immunofluorescence images (n = 3 per group). Data are presented as mean ± s.e.m. Statistical significance was assessed using the log‐rank (Mantel–Cox) test (E), two‐tailed Student's *t* test (two groups), or one‐way ANOVA (multiple groups). **p < 0.01; *** p < 0.001; ****p < 0.0001.

Pharmacological blockade of pyroptosis in the orthotopic PDAC model further confirmed the functional requirement of this pathway. Inhibition of pyroptosis significantly compromised the survival benefit induced by EP+CaCGP and led to a marked increase in tumor burden (Figure 10C–E), consistent with the CASP1‐knockdown results. Importantly, pyroptosis inhibition also largely abrogated EP+CaCGP‐induced immune activation: intratumoral CD8^+^ T‐cell infiltration was reduced, dendritic cell maturation was impaired, and macrophage polarization shifted back toward a pro‐tumorigenic phenotype (Figure 10F,G). Addition of a pyroptosis inhibitor partially attenuated EP+CaCGP‐induced upregulation of pyroptosis‐related proteins and partially restored Ki‐67 expression in tumor tissues (Figure S13D–G), further supporting the involvement of pyroptotic signaling in EP+CaCGP‐mediated tumor suppression.

Together, these loss‐of‐function experiments demonstrate that pyroptosis is not merely a correlative marker of EP+CaCGP‐induced tumor‐cell injury, but a functional mediator that links mitochondrial Ca^2+^ overload to antitumor immune remodeling. By converting mitochondrial stress into immunogenic signals, CASP1‐dependent pyroptosis contributes to CD8^+^ T‐cell infiltration, myeloid‐cell activation, and the overall therapeutic efficacy of the EP+CaCGP strategy.

### 2.8 EP+CaCGP Promotes Pro‐Inflammatory Macrophage Reprogramming by Suppressing the SPP1–CD44 Communication Axis

Single‐cell transcriptomic analysis revealed a marked increase in M1‐like macrophages in the EP+CaCGP group, suggesting that macrophage reprogramming may represent a critical component of the antitumor response induced by this regimen. Consistent with this possibility, macrophage depletion in immunocompetent mice largely abolished the tumor‐suppressive effect of EP+CaCGP, as reflected by restored tumor burden (Figure 11A). These findings indicate that macrophages are not merely associated with the remodeled immune landscape but are functionally required for the antitumor efficacy of EP+CaCGP.

**FIGURE 11:**
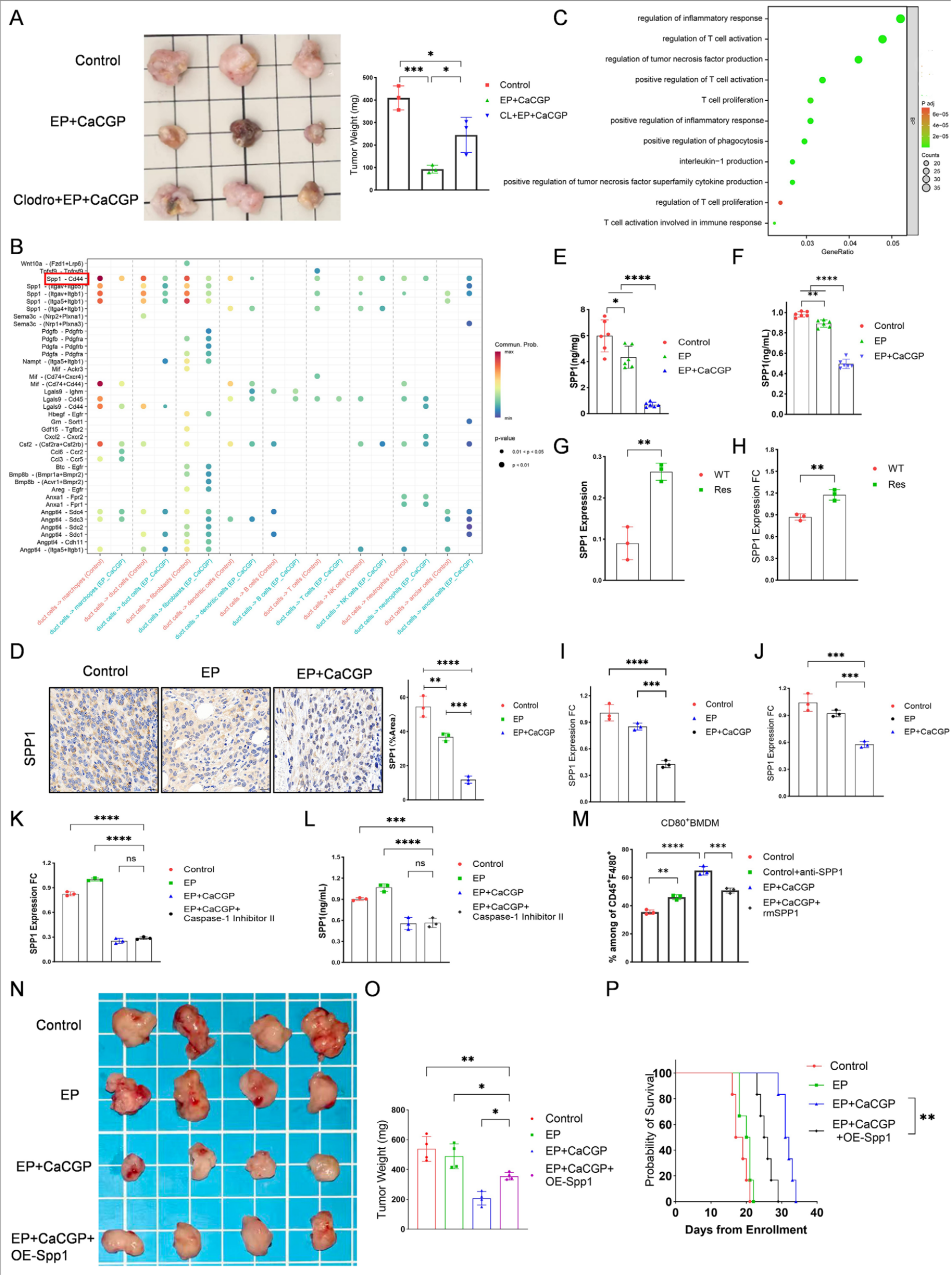
EP+CaCGP promotes pro‐inflammatory macrophage reprogramming by suppressing the SPP1–CD44 communication axis. (A) Representative tumor images and quantification of tumor weight showing that macrophage depletion (clodronate; CL) attenuates the tumor‐suppressive effect of EP+CaCGP in immunocompetent mice (n = 3 per group). EP refers to electroporation; CGP refers to mPEG‐PCL@CGP37157 micelles; Ca refers to exogenous Ca^2+^ supplementation; CaCGP refers to the combined local delivery of Ca^2+^ and CGP37157‐loaded micelles; and EP+CaCGP refers to electroporation followed by local co‐delivery of Ca^2+^ and CGP37157‐loaded micelles. (B) Cell–cell communication analysis of scRNA‐seq data (dot plot) showing ligand–receptor interaction probabilities between tumor cells and macrophages, highlighting reduced SPP1–CD44 signaling in the EP+CaCGP group (n = 3 per group). (C) GO analysis of macrophage transcriptional programs in the EP+CaCGP group, indicating enhanced inflammatory response and T‐cell activation/proliferation–associated pathways. (D) Representative immunohistochemical staining of SPP1 in tumors from the indicated groups and quantification of SPP1 positivity (n = 3 per group; scale bar = 20 um). (E) Intratumoral SPP1 levels measured by ELISA in tumors from the indicated groups (n = 6 per group). (F) SPP1 concentration in tumor cell culture supernatants measured by ELISA in Panc‐1 after the indicated treatments (n = 6 per group). (G, H) Increased SPP1 expression in EP‐resistant (Res) cells compared with wild‐type (WT) cells at the mRNA level in Panc‐1 according to bulk RNA‐seq and RT‐qPCR (n = 3 per group). (I, J) Genetic silencing of NCLX reduces SPP1 expression at the mRNA level under EP conditions in Panc‐1 and KPC‐A548 (n = 3 per group). (K, L) Pharmacological inhibition of pyroptosis (Caspase‐1 Inhibitor II) does not alter the reduction of SPP1 caused by NCLX knockdown, as shown by SPP1 mRNA expression in Panc‐1 (K) and KPC‐A548 cells (L) (n = 3 per group). (M) Tumor cell–macrophage co‐culture assay showing that SPP1 modulates macrophage polarization: anti‐SPP1 promotes, whereas recombinant SPP1 (rSPP1) counteracts, M1‐like polarization (CD80^+^ BMDMs among CD45^+^F4/80^+^ cells) (n = 3 per group). (N) Representative tumor images from orthotopic models showing that Spp1 overexpression (OE‐Spp1) partially counteracts EP+CaCGP‐mediated tumor control (n = 4 per group). (O) Tumor weight quantification for the groups in (N) (n = 4 per group). (P) Kaplan–Meier survival curves showing that the EP+CaCGP group exhibited better survival benefit than the EP+CaCGP+OE‐Spp1 group (n = 6 per group). Data are presented as mean ± s.e.m. Statistical significance was assessed using the log‐rank (Mantel–Cox) test (P), two‐tailed Student's *t* test (two groups), or one‐way ANOVA (multiple groups). *p < 0.05; **p < 0.01; ***p < 0.001; ****p < 0.0001.

To identify the tumor–macrophage communication programs involved in this macrophage‐dependent response, we interrogated the single‐cell transcriptomic data using cell–cell communication analysis. EP+CaCGP markedly attenuated the SPP1–CD44 signaling axis between tumor cells and macrophages (Figure 11B). In parallel, tumor‐associated macrophages from EP+CaCGP‐treated tumors displayed a more pro‐inflammatory transcriptional phenotype, accompanied by enhanced signaling programs associated with T‐cell activation and proliferation (Figure 11C). Cell‐type‐resolved expression analysis further showed that SPP1 was predominantly expressed by tumor cells and macrophages, whereas its receptor CD44 was mainly enriched in macrophages (Figure S14A,B), supporting SPP1–CD44 signaling as a major route of tumor cell–macrophage crosstalk in this model.

The reduction in SPP1–CD44 signaling was further supported by validation at both tissue and cellular levels. IHC analysis showed substantially decreased SPP1 expression in EP+CaCGP‐treated tumors compared with controls (Figure 11D), and ELISA confirmed reduced intratumoral SPP1 abundance after EP+CaCGP treatment (Figure 11E). Consistently, EP+CaCGP‐treated tumor cells secreted markedly lower levels of SPP1 into the culture supernatant in vitro (Figure 11F). These data identify SPP1 downregulation as a consistent molecular feature of EP+CaCGP treatment.

Mechanistically, SPP1 regulation appeared to be linked to NCLX inhibition rather than pyroptosis. EP‐resistant tumor cells exhibited elevated SPP1 expression together with increased NCLX levels (Figure 11G,H), whereas genetic silencing of NCLX significantly reduced both SPP1 mRNA expression (Figure 11I,J). By contrast, pharmacological inhibition of pyroptosis did not affect SPP1 mRNA (Figure 11K,L), indicating that SPP1 suppression occurs independently of the pyroptotic pathway. These results suggest that SPP1 downregulation represents a parallel downstream consequence of NCLX inhibition rather than a secondary effect of pyroptosis.

Functionally, tumor‐derived SPP1 directly promoted immunosuppressive macrophage polarization. In tumor cell–macrophage co‐culture assays, recombinant SPP1 robustly drove macrophages toward an M2‐like phenotype, whereas neutralization of SPP1 in tumor cell–conditioned medium substantially attenuated M2 polarization and shifted macrophages toward a more pro‐inflammatory M1‐like state (Figure 11M). These findings support a model in which tumor‐derived SPP1 acts as a paracrine driver of macrophage‐associated immunosuppression, while EP+CaCGP relieves this suppressive communication by reducing SPP1 availability and weakening SPP1–CD44 signaling.

The functional relevance of this axis was further confirmed in vivo. Overexpression of Spp1 in KPC cells partially counteracted the antitumor effects of EP+CaCGP, resulting in increased tumor burden and reduced overall survival (Figure 11N–P). Multicolor immunofluorescence analysis further showed that Spp1 overexpression partially reversed EP+CaCGP‐induced M1‐like macrophage polarization (Figure S14C). Thus, restoration of SPP1 is sufficient to blunt both macrophage reprogramming and therapeutic efficacy.

These findings support a model in which EP+CaCGP promotes antitumor macrophage reprogramming through a pyroptosis‐independent NCLX–SPP1–CD44 regulatory axis. Although the precise molecular mechanism by which NCLX controls SPP1 expression remains to be defined, NCLX inhibition reduces tumor‐derived SPP1, weakens SPP1–CD44‐mediated tumor cell–macrophage crosstalk, alleviates macrophage‐associated immunosuppression, and thereby enhances the in vivo antitumor efficacy of EP+CaCGP.

### 2.9 In Vivo Biosafety Evaluation of the EP+CaCGP Combination Regimen

Given the potent antitumor activity of EP+CaCGP, we next performed a systematic in vivo biosafety assessment to define its potential translational applicability. At a predefined time point during the treatment course (day 4), major organs (heart, liver, lung, kidney, and small intestine) were harvested and subjected to histopathological examination by hematoxylin and eosin (H&E) staining. Microscopic analysis revealed that, compared with controls, all examined organs from EP+CaCGP‐treated mice preserved intact tissue architecture, with no evident signs of cellular necrosis, inflammatory cell infiltration, or fibrosis (Figure 12A), indicating an absence of overt target‐organ toxicity.

**FIGURE 12:**
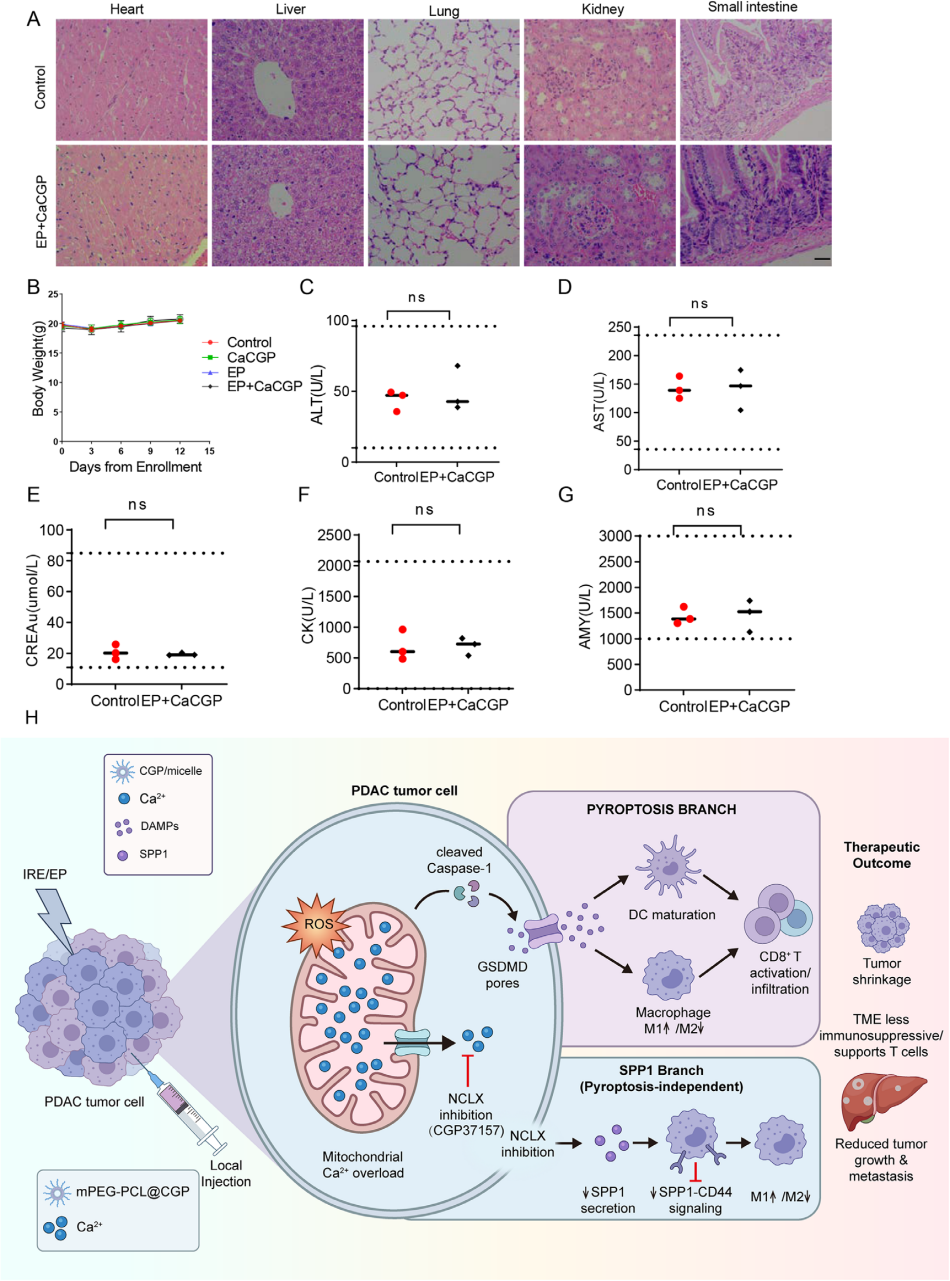
In vivo biosafety evaluation of the EP+CaCGP combination regimen and abstract figure. (A) Representative histopathological images of major organs (heart, liver, lung, kidney, small intestine) from control and EP+CaCGP‐treated mice, stained with hematoxylin and eosin (H&E). No signs of cellular necrosis, inflammatory cell infiltration, or fibrosis were observed in EP+CaCGP‐treated tissues (scale bar = 50 µm). (B) Body weight monitoring over the course of treatment, showing stable body weight in EP+CaCGP‐treated mice compared to controls (n = 3 per group). (C) Serum ALT levels in control and EP+CaCGP‐treated mice, showing no significant differences (n = 3 per group). (D) Serum AST levels in control and EP+CaCGP‐treated mice, indicating no significant differences (n = 3 per group). (E) Serum creatinine (CREA) levels in control and EP+CaCGP‐treated mice, showing no significant changes (n = 3 per group). (F) Serum creatine kinase (CK) levels in control and EP+CaCGP‐treated mice, with no significant differences (n = 3 per group). (G) Serum amylase (AMY) activity in control and EP+CaCGP‐treated mice, showing no significant alterations (n = 3 per group). (H) Schematic illustration of the proposed mechanisms underlying the antitumor effects of EP+CaCGP. Created using Figdraw with copyright authorization (ID: IUTOR8aaa8). Data are presented as mean ± s.e.m. Statistical significance was assessed using the log‐rank (Mantel–Cox) test, two‐tailed Student's *t* test, or one‐way ANOVA. *ns, not significant*.

In parallel, we monitored body‐weight dynamics throughout treatment as a gross indicator of systemic well‐being. EP+CaCGP‐treated mice maintained stable body weight without significant fluctuations or growth retardation (Figure 12B). To sensitively capture potential biochemical toxicities, we further measured key serum parameters, including liver function markers (ALT and AST), renal function (creatinine), a marker of myocardial injury (CK), and pancreatic enzyme activity (amylase). All indices remained within physiological ranges in the treatment group and were not statistically different from those in healthy controls (Figure 12C–G), arguing against acute hepatotoxicity, nephrotoxicity, cardiac injury, or treatment‐associated pancreatitis.

Collectively, these data demonstrate favorable biocompatibility and short‐term safety of the EP+CaCGP combination in mice, with no observable systemic adverse effects or major organ toxicities, thereby providing a safety foundation for further preclinical development. The schematic diagram of the proposed mechanism is shown in Figure 12H.

## 3 Discussion

Our study identifies NCLX‐mediated mitochondrial Ca^2+^ extrusion as a key adaptive mechanism that enables PDAC cells to survive irreversible electroporation and acquire treatment resistance. After EP‐induced membrane permeabilization and Ca^2+^ influx, residual tumor cells upregulate NCLX to limit mitochondrial Ca^2+^ overload and avoid lethal injury. Targeting this vulnerability, we developed the EP+CaCGP strategy, which combines NCLX inhibition with exogenous Ca^2+^ delivery during the EP window to amplify mitochondrial Ca^2+^ overload. This regimen induces mitochondrial dysfunction, redox imbalance, DAMP release, and GSDMD‐dependent pyroptosis, thereby enhancing antitumor immunity. In parallel, EP+CaCGP downregulates tumor‐derived SPP1 and weakens SPP1–CD44‐mediated tumor–macrophage crosstalk, promoting pro‐inflammatory myeloid reprogramming. Given the electric‐field gradients in clinical ablation zones and the persistence of recurrence‐prone cells in low‐field margins, we use “EP” as a unified framework to emphasize therapeutic intervention against marginal residual tumor cells and their immunosuppressive microenvironment.

From a translational standpoint, IRE is being explored as a local control strategy for selected patients with locally advanced or unresectable PDAC, particularly for tumors adjacent to major vessels where thermal ablation is limited. However, IRE is not intended to replace surgery, chemotherapy, or radiotherapy, and in many clinical settings it remains an investigational or selectively applied approach rather than a routine standard of care [24]. Although IRE has been adopted in selected centers, recurrence and distant dissemination continue to limit durable benefit. This likely reflects both the regenerative potential of residual cells in low‐field margins and the intrinsically immunosuppressive PDAC microenvironment [25, 26]. Within the “seed–soil” paradigm [15, 16, 17], our findings suggest that mitochondrial Ca^2+^ homeostatic adaptation after EP not only supports residual tumor‐cell survival as a “seed” of recurrence, but may also influence the immunological consequences of cell death and paracrine signaling within the “soil”. Thus, the NCLX–mitochondrial Ca^2+^ extrusion axis provides a shared therapeutic entry point linking residual‐cell survival and immune suppression.

Mechanistically, EP‐driven ionic imbalance promotes rapid Ca^2+^ entry and mitochondrial uptake [19, 20]. Although mitochondrial injury increases with Ca^2+^ load, some cells remain below the death threshold and acquire a resistant phenotype. As a major route for mitochondrial Ca^2+^ efflux, NCLX acts as a threshold‐control mechanism [21]. Genetic silencing of NCLX markedly increases EP sensitivity, whereas NCLX overexpression is protective, supporting NCLX as a causal determinant of cell fate after EP. By coupling pharmacological NCLX inhibition with Ca^2+^ provision, CaCGP “locks in” mitochondrial Ca^2+^ overload under EP and triggers lethal mitochondrial injury.

Importantly, the therapeutic benefit of EP+CaCGP is not merely a consequence of enhanced cytotoxicity, but reflects an immunological redirection of the cell‐death spectrum. IRE/EP has often been associated with apoptosis and other relatively “low‐inflammatory” forms of death, which may provide insufficient danger signals for robust immune priming [27]. In contrast, inflammatory death programs such as pyroptosis, characterized by membrane pore formation and release of intracellular contents, can more effectively convert local tumor injury into antigen presentation and effector immune activation [28]. Our data indicate that EP+CaCGP shifts EP‐induced cell death toward inflammasome–GSDMD‐driven pyroptosis, thereby promoting antigen presentation, pro‐inflammatory myeloid polarization, and CD8^+^ T‐cell effector responses. The marked reduction in efficacy in adaptive immune‐deficient settings further indicates that the in vivo benefit depends not only on local tumor killing, but also on immune‐mediated amplification and maintenance. Within the tested parameter window, EP+CaCGP caused no overt acute organ toxicity, supporting further optimization of this strategy.

Beyond this “death modality–immune amplification” axis, EP+CaCGP also engages a parallel mechanism of “soil” reprogramming. Specifically, EP+CaCGP attenuates SPP1–CD44 communication between tumor cells and macrophages and promotes pro‐inflammatory macrophage reprogramming. This effect appears to be at least partly independent of pyroptosis, as SPP1 expression changes concordantly with NCLX activity, whereas pyroptosis blockade does not alter SPP1 transcript or protein abundance. SPP1, also known as osteopontin, has been implicated as a secreted driver of PDAC progression, with increased expression in advanced disease and functional roles in tumorigenesis, metastasis, and malignant cell‐state maintenance [30]. Immunologically, SPP1 signaling has been linked to immune exclusion and impaired effector function: the SPP1–CD44 axis can suppress CD8^+^ T‐cell proliferation and function, whereas CD44 blockade can partially restore T‐cell activity [29]. SPP1 can also remodel stromal programs through CD44 and strengthen immunosuppressive chemokine networks, thereby limiting T‐cell infiltration and reducing immunotherapy responsiveness [31]. In this context, EP+CaCGP‐mediated downregulation of tumor‐derived SPP1 provides an additional mechanism by which NCLX inhibition may weaken tumor–macrophage immunosuppressive crosstalk. Prior links between SPP1 secretion, redox homeostasis, and mitochondrial stress further suggest a testable connection between mitochondrial Ca^2+^ disequilibrium, oxidative/secretory stress, and SPP1 suppression [29].

Placed within the broader landscape of IRE/EP potentiation, existing strategies mainly include “add‐on” approaches that provide exogenous immune stimulation after EP/IRE, and “alternative‐death” approaches that induce non‐apoptotic programs such as ferroptosis to reduce residual tumor outgrowth [25, 26, 32, 33, 34]. In contrast, our work targets an early, pharmacologically actionable adaptive mechanism—NCLX‐mediated mitochondrial Ca^2+^ extrusion—and uses “forced mitochondrial Ca^2+^ overload” to connect local tumor sensitization with immune remodeling. This strategy lowers the survival threshold of resistant “seeds” while loosening myeloid immunosuppressive crosstalk within the “soil”.

Several directions warrant further study. First, although we establish the functional importance of NCLX in EP resistance and SPP1‐mediated macrophage reprogramming, the mechanisms by which EP induces NCLX upregulation and by which NCLX inhibition downregulates SPP1 remain unclear. Second, post‐IRE human PDAC specimens were not available because additional tissue acquisition after IRE is generally not clinically indicated and may raise ethical concerns; future studies using patient‐derived xenograft models and other human‐relevant platforms are therefore needed to validate the NCLX axis and EP+CaCGP responsiveness. Third, although EP+CaCGP improved residual tumor control, survival, and systemic antitumor immunity, the rapidly progressing models used here do not allow rigorous assessment of long‐term local recurrence; recurrence models suitable for extended follow‐up will be needed to evaluate durable tumor control. Finally, EP+CaCGP requires further translational optimization. CGP37157‐loaded micelles should be regarded as a proof‐of‐concept tool rather than a clinical candidate, and more selective NCLX‐targeting strategies with systematic off‐target controls are needed. In addition, despite local Ca^2+^ delivery in this study, longer‐term and repeat‐dose studies should evaluate potential systemic effects of exogenous Ca^2+^, including calcium‐homeostasis disturbance and cardiovascular or renal safety risks.

In summary, we define NCLX‐mediated mitochondrial Ca^2+^ extrusion as a key adaptive axis supporting PDAC residual survival and EP resistance. Combining NCLX inhibition with Ca^2+^ provision during the EP window converts this adaptation into a therapeutic vulnerability by shifting cell death toward inflammatory, immunologically consequential pyroptosis while weakening SPP1–CD44‐driven myeloid immunosuppressive communication. This dual “seed” and “soil” intervention may improve residual tumor control and enhance the therapeutic efficacy of EP‐based treatment.

## 4 Methods

### 4.1 Cell Culture, Authentication, and Mycoplasma Testing

The human PDAC cell line Panc‐1 used in this study was purchased from the Cell Resource Center, Institute of Basic Medical Sciences, Chinese Academy of Medical Sciences and Peking Union Medical College. The murine pancreatic cancer cell line KPC‐A548 was established in our laboratory from an orthotopic pancreatic cancer model generated using LSL‐Kras^G12D/+; LSL‐Trp53^R172H/+; Pdx‐1‐Cre (KPC) mice, followed by isolation and primary culture of tumor tissues. All cell lines were authenticated by short tandem repeat (STR) profiling to confirm their identity and origin. Cell culture was performed under strict aseptic conditions, and routine mycoplasma testing was conducted during the experiments, with all results confirmed to be negative.

KPC‐A548 and Panc‐1 cells were cultured in complete Dulbecco's Modified Eagle's Medium (DMEM) supplemented with 10% fetal bovine serum (FBS) and 1% penicillin–streptomycin–amphotericin B.

### 4.2 Human PDAC Specimens

Fresh PDAC tissues (n = 3) were obtained from the Department of Pancreatic Surgery at Wuhan Union Hospital (Wuhan, China). Sample collection and downstream use were approved by the institutional ethics committee (Approval Number: 20250476). All tissues were processed immediately and used for the establishment of patient‐derived PDAC organoids. Where applicable, informed consent was obtained in accordance with institutional guidelines and the Declaration of Helsinki.

### 4.3 Animals

Eight‐week‐old female C57BL/6 mice and nude mice were purchased from Wuhan Beiente Biotechnology Co., Ltd. and maintained under specific pathogen–free (SPF) conditions at the Animal Center of Tongji Medical College, Huazhong University of Science and Technology. All animal experiments were approved by the Institutional Animal Care and Use Committee of Tongji Medical College, Huazhong University of Science and Technology (approval No. 20234505).

### 4.4 Western Blot Analysis

Pancreatic cancer cells subjected to low‐intensity electroporation (EP) were collected for Western blotting. Briefly, cell suspensions were adjusted to 5 × 10^5^ cells/mL, and 700 µL of suspension was transferred to an electroporation cuvette and exposed to 600 V/cm, 20 pulses. Cells were then seeded into 6‐well plates (3.5 × 10^5^ cells/well) and cultured for 24 h. After washing once with PBS, cells were lysed in RIPA buffer supplemented with protease inhibitors, phosphatase inhibitors, and PMSF. Lysates were collected on ice, sonicated (35 W, 1 s on/1 s off, total 1 min), and centrifuged at 13,000 rpm, 4°C for 30 min. Protein concentration was determined by the BCA assay, and 20–30 µg protein per lane was mixed with 5× loading buffer (4:1, v/v) and denatured at 95°C for 10 min.

Proteins were separated on 10%–12% SDS–PAGE gels and transferred onto PVDF membranes (activated in methanol for 1 min) using wet transfer at 160 V for 70 min on ice. Membranes were blocked with 5% non‐fat milk in TBST for 1 h at room temperature, incubated with primary antibodies overnight at 4°C, followed by TBST washes and incubation with HRP‐conjugated secondary antibodies for 1 h at room temperature. Signals were visualized using ECL chemiluminescence and captured with a gel imaging system. Antibody information is provided in Supplementary Table S1, and related experimental materials are listed in Table S2. For quantification, band intensities can be measured in ImageJ and normalized to a loading control (e.g., β‐actin).

### 4.5 Immunohistochemistry (IHC) and H&E Staining

#### 4.5.1 Hematoxylin and Eosin (H&E) Staining

Paraffin sections were baked, deparaffinized, and rehydrated as described above. Sections were stained with hematoxylin for 5 min, rinsed in distilled water, differentiated briefly in 0.4% hydrochloric acid (∼2 s), and washed under running water for 30 min. Sections were then stained with eosin for 5 min, dehydrated through graded ethanol (70%, 95%, 100%, 5 min each), cleared in xylene (two changes, 15 min each), and mounted with neutral resin. Slides were examined under a bright‐field microscope.

#### 4.5.2 Immunohistochemistry

At indicated time points, tumors and major organs were harvested, fixed in 4% paraformaldehyde for 24 h, and processed for paraffin embedding and sectioning by a commercial service provider (Wuhan Servicebio, China). Paraffin sections were baked at 60°C for 30 min, deparaffinized in xylene (two changes, 5 min each), and rehydrated through graded ethanol (100%, 95%, 70%, 3 min each) to water. Antigen retrieval was performed in sodium citrate buffer (pH 6.0) by heating at 95°C for 30 min, followed by cooling to room temperature. Sections were washed in TBST (3 × 5 min).

Endogenous peroxidase activity was quenched with 3% H_2_O_2_ for 10 min at room temperature (protected from light), followed by TBST washes (3 × 5 min). Sections were blocked with 5% BSA for 1 h at room temperature in a humidified chamber, incubated with primary antibodies (diluted in blocking buffer) overnight at 4°C, washed with TBST (3 × 5 min), and then incubated with HRP‐conjugated secondary antibody for 1 h at room temperature. Signal was developed using DAB substrate with reaction time monitored under a bright‐field microscope and stopped by immersion in water.

Nuclei were counterstained with hematoxylin for 5 min, blued in 0.1% ammonia water for 3 min, and rinsed in distilled water. Sections were dehydrated through graded ethanol (70%, 95%, 100%, 5 min each), cleared in xylene (two changes, 15 min each), and mounted with neutral resin. Images were acquired by light microscopy, and positive staining was quantified (e.g., percent positive area/cells) using ImageJ or equivalent software. Information on the antibodies used in this study is provided in Table S1.

#### 4.5.3 Tissue Immunofluorescence (IF)

Tissue immunofluorescence staining followed the same procedures as IHC through primary antibody incubation (including deparaffinization/rehydration, antigen retrieval, and blocking). After incubation with primary antibodies overnight at 4°C, sections were washed in TBST and incubated with species‐matched fluorophore‐conjugated secondary antibodies for 1 h at room temperature in the dark. Sections were washed with TBST (3 × 5 min) and counterstained with freshly prepared DAPI for 10 min at room temperature in the dark. Slides were mounted using an anti‐fade mounting medium and imaged by fluorescence microscopy. Information on the antibodies used in this study is provided in Table S1 in the Supplementary Information.

#### 4.5.4 RT–qPCR Analysis

Total RNA was extracted from cells or tissues according to the manufacturer's instructions (e.g., TRIzol‐based or column‐based kits), followed by assessment of RNA concentration and purity. Equal amounts of RNA were reverse‐transcribed into cDNA. Quantitative PCR was performed using a SYBR Green master mix on a real‐time PCR system. Technical replicates (typically 2–3 per sample) and a no‐template control (NTC) were included. Target gene expression was normalized to a housekeeping gene (ACTB), and relative expression levels were calculated using the 2^−ΔΔCt^ method. Primer sequences are provided in the Supplementary Information (e.g., Table S3).

#### 4.5.5 CCK‐8 Cell Viability Assay

Cell viability after electroporation was quantified using a Cell Counting Kit‐8 (CCK‐8) assay (Dojindo), following the manufacturer's instructions with minor modifications. Briefly, pancreatic cancer cells were resuspended at 5 × 10^5^ cells/mL, and 700 µL of cell suspension was transferred into 4‐mm gap electroporation cuvettes. Cells were subjected to electroporation at 600 V/cm for 20 pulses (pulse width and frequency as specified in the electroporation section), or as indicated for parameter‐screening experiments (electric field strength 0–800 V/cm, with or without CGP37157 and/or CaCGP). Immediately after treatment, cells were diluted in complete culture medium and seeded into 96‐well plates at 1 × 10^4^ cells per well (100 µL per well). Plates were incubated at 37°C with 5% CO_2_ for 24 h.

Subsequently, culture medium was removed and 60 µL of CCK‐8 working solution was added to each well. After incubation at 37°C for 1–2 h, absorbance was measured at 450 nm using a microplate reader. Background absorbance from blank wells (medium plus CCK‐8 without cells) was subtracted, and cell viability was expressed as normalized absorbance relative to the corresponding control group.

#### 4.5.6 Immunofluorescence Staining and Confocal Microscopy

Glass coverslips were sterilized by immersion in 75% ethanol overnight, air‐dried, and placed in 12‐well plates. Pancreatic cancer cells were subjected to electroporation as indicated (e.g., 600 V/cm, 20 pulses; or voltage 0–800 V/cm; ±CGP37157 and/or ±CaCGP), then seeded onto coverslips at 1 × 10^5^ cells per well and cultured at 37°C for 24 h.

Cells were washed once with PBS and fixed with 4% paraformaldehyde for 20 min at room temperature, followed by PBS washing. Cells were permeabilized with 0.5% Triton X‐100 for 15 min at room temperature, washed with PBS, and blocked with 1% BSA for 30 min at room temperature. Coverslips were then incubated with primary antibodies overnight at 4°C, washed three times with TBST (10 min each), and incubated with fluorophore‐conjugated secondary antibodies for 1 h at room temperature in the dark. After three additional TBST washes (10 min each), nuclei were counterstained with Hoechst for 10 min at room temperature, followed by TBST washing.

Coverslips were mounted using an anti‐fade mounting medium (10 µL per slide), sealed, and imaged using a laser‐scanning confocal microscope. Acquisition settings were kept constant within each experiment.

#### 4.5.7 LDH Release Assay

Pancreatic cancer cells were subjected to electroporation as indicated (e.g., 600 V/cm, 20 pulses; or field strength 0–800 V/cm; ±CGP37157 and/or ±CaCGP), diluted, and seeded into 96‐well plates at 1 × 10^4^ cells per well. After 24 h incubation at 37°C, culture supernatants were collected, and lactate dehydrogenase (LDH) activity was measured using a commercial LDH assay kit according to the manufacturer's instructions. Briefly, 60 µL of LDH working solution was added per well, samples were incubated for 30 min at room temperature protected from light, and absorbance was read at 490 nm using a microplate reader. LDH release was calculated per kit instructions. The relevant experimental materials are listed in supplementary Table S2.

#### 4.5.8 Extracellular ATP Assay (Cell Culture Supernatant)

Cells were treated by electroporation as indicated, diluted, and seeded into 96‐well plates at 2 × 10^4^ cells per well. After 24 h, supernatants were collected and clarified by centrifugation (12,000 × g, 5 min, 4°C). Cleared supernatants were transferred to white opaque 96‐well plates. ATP standards and detection working solution were prepared following the manufacturer's instructions. To reduce background, 100 µL of ATP detection working solution was pre‐dispensed into a fresh white plate and incubated at room temperature for 5 min. A total of 20 µL of each sample or standard was then added, mixed, and luminescence (RLU) was recorded using a multimode plate reader with a luminometer function (reads initiated at 5‐s intervals). ATP concentrations were calculated from the standard curve. The relevant experimental materials are listed in supplementary Table S2.

#### 4.5.9 Mitochondrial Ca^2+^ Measurement (Rhod‐2 AM)

After electroporation as indicated, cells were diluted and plated in 24‐well plates at 5 × 10^4^ cells per well and cultured for 24 h. Rhod‐2 AM staining solution was prepared according to the manufacturer's instructions. Cells were washed once with PBS and incubated with 300 µL Rhod‐2 AM working solution per well for 30 min at 37°C. Cells were then washed twice with PBS and incubated in complete medium containing serum before imaging. Fluorescence signals were acquired using a laser‐scanning confocal microscope under consistent settings within each experiment.

#### 4.5.10 Mitochondrial Membrane Potential (JC‐1)

Cells were electroporated as indicated, diluted, and seeded in 24‐well plates at 5 × 10^4^ cells per well for 24 h. JC‐1 staining solution was prepared per the manufacturer's protocol. Cells were washed once with PBS, then incubated with JC‐1 staining solution (500 µL culture medium + 500 µL JC‐1 staining solution per well) for 20 min at 37°C. After staining, cells were washed twice with JC‐1 staining buffer and maintained in 1 mL culture medium for imaging. JC‐1 fluorescence was imaged by confocal microscopy, and mitochondrial depolarization was assessed based on the relative distribution of JC‐1 aggregates (red) versus monomers (green) using identical acquisition settings across groups.

#### 4.5.11 Induction of EP‐Resistant Cell Line

Electric pulses were generated using an HW‐E01 electroporation system (Huiwei Medical, Ningbo, China). For in vitro experiments, pulses were applied to cell suspensions loaded in electroporation cuvettes equipped with two aluminum plate electrodes spaced 4 mm apart (Cat. No. 1652081, Bio‐Rad, Hercules, CA, USA). Panc‐1 and KPC‐A548 cell suspensions were electroporated at an initial field strength of 500 V/cm with a pulse duration of 100 µs, a repetition frequency of 1 Hz, and 99 pulses. Surviving cells were allowed to expand from single‐cell clones to confluence, after which the electric‐field strength was increased, and cells were re‐challenged, with the field strength raised by 100 V/cm at each round. The induction was considered complete when cells tolerated 1400 V/cm with 99 pulses while maintaining ≤20% cell death. Under the commonly used electroporation condition for routine assays (600 V/cm, 100 µs, 1 Hz, 99 pulses), the EP‐resistant cells consistently showed >90% viability, whereas control (parental) cells exhibited <30% viability.

#### 4.5.12 siRNA‐Mediated Knockdown of SLC8B1 in Panc‐1 Cells

Panc‐1 cells were seeded in 6‐well plates (1 × 10^5^ cells per well) and cultured for 24 h to reach ∼30%–50% confluence. For transfection, 5 µL of 20 µM siRNA (final concentration 50 nM) was diluted in 250 µL Opti‐MEM and mixed gently. In parallel, 2 µL Lipo8000 was diluted in 250 µL Opti‐MEM and incubated for 5 min at room temperature. The diluted siRNA and transfection reagent were then combined and incubated for 20 min at room temperature to allow complex formation.

Culture medium was replaced with 1.5 mL fresh complete medium per well, and 500 µL of the transfection complexes were added dropwise to each well with gentle rocking. Knockdown efficiency was assessed 72 h post‐transfection by collecting cells for protein extraction and immunoblotting. The gene knockdown sequences are provided in Supplementary Table S4.

#### 4.5.13 Generation of Stable Slc8b1‐Knockdown, and Casp1‐Knockdown KPC A548 Cells by Lentiviral Transduction

KPC A548 cells were seeded in 6‐well plates at a density of 5 × 10^4^ cells per well and cultured for 24 h until they reached approximately 20–30% confluence. Cells were then transduced with lentiviral particles carrying murine Slc8b1‐targeting shRNA and murine Casp1‐targeting shRNA constructs at the indicated multiplicity of infection (MOI). Corresponding negative‐control shRNA or empty‐vector lentiviral particles were used as controls. The volume of lentivirus added to each well was calculated according to the following formula:

VirusVolume=MOI×CellNumber/ViralTiter

After 24 h of incubation at 37°C, the viral supernatant was replaced with fresh complete medium, and cells were further cultured. At 72 h post‐transduction, transduction efficiency was assessed by fluorescence microscopy, and the MOI was empirically optimized based on the observed fluorescence‐positive cell proportion and cell viability.

To establish stable cell lines, transduced cells were selected with puromycin at 15 µg/mL. The selection medium was refreshed every 48 h, and cell morphology and viability were monitored throughout the selection period. Non‐transduced control cells were completely eliminated within 4–6 days. After 6 days of puromycin selection, surviving cells were expanded for downstream experiments. Knockdown efficiency of Slc8b1 and Casp1 was validated by qRT‐PCR and/or immunoblotting. The stable cell populations showing the most efficient target gene knockdown or overexpression were used for subsequent experiments. The construction of the above lentiviral vectors was commissioned to Shanghai Genechem Co., Ltd. The shRNA target sequences are provided in Supplementary Table S5.

#### 4.5.14 Plasmid‐Mediated Overexpression of Slc8b1

KPC A548 cells were seeded in 6‐well plates (2 × 10^5^ cells per well) in antibiotic‐free complete medium and transfected when confluence exceeded 70%. For each well, 2 µg pLVX‐Slc8b1‐Flag‐Puro plasmid was diluted in 250 µL Opti‐MEM, and Neofect transfection reagent (2 µL) was diluted separately in 250 µL Opti‐MEM. After a 5‐min incubation at room temperature, the two solutions were combined, mixed gently, and incubated for 20 min at room temperature to allow DNA–lipid complex formation. The complexes (500 µL per well) were added dropwise to cells with gentle rocking. After ∼16 h, the medium was replaced with fresh complete medium. Puromycin selection (15 µg/mL) was initiated the next day (medium refreshed as needed). Slc8b1 overexpression was verified by immunoblotting prior to downstream experiments. The construction of the above plasmids was commissioned to Shanghai Genechem Co., Ltd.

#### 4.5.15 shRNA Plasmid‐Mediated Knockdown of NLRP3 and GSDMD

For gene knockdown, shRNA plasmids targeting murine Nlrp3 or Gsdmd and human NLRP3 or GSDMD were used according to the species origin of the corresponding PDAC cell lines. A non‐targeting shRNA plasmid served as the negative control. Murine KPC A548 cells or human PDAC cells were seeded into 6‐well plates at 2 × 10^5^ cells per well in antibiotic‐free complete medium and transfected when the cells reached more than 70% confluence. For each well, 2 µg shRNA plasmid was diluted in 250 µL Opti‐MEM, and 2 µL Neofect transfection reagent was diluted separately in 250 µL Opti‐MEM. After a 5‐min incubation at room temperature, the plasmid and transfection reagent solutions were combined, gently mixed, and incubated for 20 min at room temperature to form DNA–lipid complexes. The complexes were added dropwise to the cells, followed by gentle rocking to ensure even distribution. Approximately 16 h after transfection, the medium was replaced with fresh complete medium. The construction of the above plasmids was commissioned to Shanghai Genechem Co., Ltd., and the gene knockdown sequences are provided in Supplementary Table S4.

### 4.6 Preparation and Characterization of mPEG–PCL@CGP37157 Micelles

#### 4.6.1 Preparation of CGP37157‐Loaded Micelles (mPEG–PCL@CGP37157)

CGP37157 was dissolved in dimethyl sulfoxide (DMSO) by sonication to prepare a 50 mg mL^−^^1^ stock solution. For micelle fabrication, 100 µL of the CGP37157 stock was used. mPEG–PCL (200 mg) was dissolved in 2 mL acetone under sonication until the solution became clear. The CGP37157/DMSO solution (100 µL) was added to the polymer solution and mixed at room temperature for 1 h to yield a homogeneous organic phase.

Micelles were formed by solvent displacement. The organic phase was transferred to a 10‐mL centrifuge tube, and 2 mL ultrapure water was rapidly added under vigorous vortexing, followed by vortexing for an additional 1 min. Acetone was removed under reduced pressure at 45°C and 100 mBar for 10 min. The resulting dispersion was dialyzed using a 3.5‐kDa MWCO dialysis membrane against ultrapure water at 4°C for 2 h. After centrifugation at 3000 rpm for 5 min to remove insoluble aggregates, the supernatant was collected and sterile‐filtered through a 0.22‐µm syringe filter to obtain mPEG–PCL@CGP37157 micelles.

#### 4.6.2 Quantification of Drug Loading by HPLC

To quantify encapsulated CGP37157, the micelle suspension was mixed with DMSO at a 1:9 (v/v) ratio, sonicated for 10 min, and filtered through a 0.22‐µm syringe filter. CGP37157 was quantified by high‐performance liquid chromatography (HPLC) using mobile phase A: 0.1% (v/v) trifluoroacetic acid in water and mobile phase B: acetonitrile, at a flow rate of 1.0 mL min^−^^1^. The gradient program was 0 min, 90% A/10% B; 10 min, 10% A/90% B; 20 min, 90% A/10% B. 20 µL of sample was injected per run. Drug loading was calculated from CGP37157 peak areas using an external standard calibration curve.

#### 4.6.3 In Vitro Drug Release

In vitro release of CGP37157 from micelles was evaluated by dialysis in buffers mimicking physiological and acidic conditions. Phosphate‐buffered saline (PBS, pH 7.4) and acetate‐adjusted PBS (pH 5.2) were prepared. Micelle suspension (400 µL) was mixed with 400 µL of the corresponding buffer, loaded into SpectraPor Float‐A‐Lyzer G2 dialysis devices (3.5‐kDa MWCO), and dialyzed against the corresponding release medium at 37°C with agitation. At 0, 2, 4, 6, 12, 24, and 48 h, aliquots (20 µL) were withdrawn and immediately mixed with 180 µL DMSO, then stored at 4°C until analysis. Samples were filtered through a 0.22‐µm syringe filter, and 20 µL was injected into HPLC for quantification. Cumulative release profiles were calculated accordingly.

#### 4.6.4 Dynamic Light Scattering (DLS) for Hydrodynamic Size Measurement

Micelles were diluted 1:10 (v/v) in double‐distilled water. Hydrodynamic diameter was measured by dynamic light scattering (DLS) by loading 2 mL of diluted sample into a disposable cuvette, following the manufacturer's standard measurement protocol.

#### 4.6.5 Bone Marrow–Derived Dendritic cells (BMDCs)

Bone marrow–derived dendritic cells (BMDCs) were generated from 8‐week‐old C57BL/6 mice under sterile conditions. Briefly, femurs and tibias were harvested, briefly surface‐sterilized in 70% ethanol, transferred to cold sterile PBS, and the epiphyses were removed. Bone marrow was flushed using cold complete RPMI‐1640 (RPMI‐1640 supplemented with 10% fetal bovine serum and 1% penicillin/streptomycin), gently dissociated to obtain a single‐cell suspension, and passed through a 70‐µm cell strainer. Cells were pelleted (400 × g, 5 min), resuspended, counted, and seeded at 2 × 10^6^ viable cells per 10‐cm dish in 10 mL complete RPMI‐1640 supplemented with 20 ng/mL GM‐CSF. Cultures were maintained at 37°C with 5% CO_2_. On day 3, 10 mL of fresh GM‐CSF–containing medium was added. On day 6, half of the culture medium was replaced with fresh GM‐CSF–containing medium. On day 8, non‐adherent and loosely adherent cells were gently collected and used as BMDCs for subsequent experiments.

#### 4.6.6 Bone Marrow–Derived Macrophages (BMDMs)

Bone marrow–derived macrophages (BMDMs) were generated from bone marrow cells prepared as described above. Cells were seeded at 2 × 10^6^ viable cells per 10‐cm dish in complete RPMI‐1640 supplemented with 10 ng/mL M‐CSF and cultured at 37°C with 5% CO_2_. Fresh M‐CSF–containing medium was replaced every 3 days. On day 7, adherent cells were harvested and used as BMDMs.

#### 4.6.7 Splenic T‐Cell Isolation

Splenic T cells were isolated from C57BL/6 mice. Spleens were mechanically dissociated to generate single‐cell suspensions. After centrifugation (4°C), red blood cells were lysed using RBC lysis buffer for 2 min on ice and quenched with PBS or RPMI‐1640. Cells were filtered, washed, and resuspended in PBS containing 2% FBS and 1 mM EDTA. Total T cells were purified by negative selection using the EasySep Mouse T Cell Isolation Kit following the manufacturer's instructions, including Fc receptor blocking and magnetic separation with RapidSpheres. Purified T cells were collected and used immediately for downstream co‐culture assays.

#### 4.6.8 In Vitro Co‐Culture Assays With Electroporated Tumor Cells

To assess immune activation following electroporation, pancreatic cancer cells were treated 24 h prior to immune‐cell collection. Tumor cells were resuspended at 5 × 10^5^ cells/mL, loaded into electroporation cuvettes, and subjected to electroporation at 600 V/cm with 20 pulses (with indicated conditions, e.g., ±CGP37157 and/or ±CaCGP). After treatment, cells were diluted and plated in 6‐well plates at 3 × 10^5^ cells per well and cultured for 24 h. BMDCs, BMDMs, and splenic T cells were then collected, counted, and adjusted to 3 × 10^5^ cells/mL. For co‐culture, half of the tumor‐cell culture medium (1 mL) was replaced with 1 mL immune‐cell suspension to establish tumor cell–BMDC or tumor cell–BMDM co‐cultures. For tri‐cultures, immune cells were combined as indicated (e.g., tumor cells + BMDCs + T cells; tumor cells + BMDMs + T cells) at predefined ratios (typically 1:1:1, unless otherwise specified). After co‐culture, immune phenotypes and activation status were evaluated by flow cytometry.

### 4.7 Flow Cytometry

#### 4.7.1 Flow Cytometric Analysis of Cell Death (Annexin V–FITC/PI)

Tumor cells were subjected to electroporation as indicated (e.g., 600 V/cm, 20 pulses; ±CGP37157 and/or ±CaCGP), diluted, seeded into 6‐well plates (3 × 10^5^ cells per well), and cultured for 24 h. Both floating and adherent cells were collected to avoid underestimation of cell death. Culture supernatants were retained, while adherent cells were detached using EDTA‐free trypsin (digestion time was minimized to reduce false‐positive staining). Cells were combined with the corresponding supernatants, pelleted (400 × g, 5 min, 4°C), and washed twice with ice‐cold PBS.

Cells were resuspended in ice‐cold 1× Binding Buffer and adjusted to 1–5 × 10^6^ cells/mL. For staining, 100 µL of cell suspension was incubated with Annexin V–FITC/PI apoptosis detection reagents (Servicebio, G1511‐100T; Annexin V–FITC, 3 µL; PI, 3 µL) for 8 min at room temperature in the dark. Subsequently, 200 µL of ice‐cold 1× Binding Buffer was added. Samples were filtered through a 300‐mesh nylon filter and analyzed by flow cytometry within 30 min. Quadrants were defined to distinguish viable (Annexin V^−^/PI^−^), early apoptotic (Annexin V^+^/PI^−^), late apoptotic/necrotic (Annexin V^+^/PI^+^), and primary necrotic (Annexin V^−^/PI^+^) populations.

#### 4.7.2 Flow Cytometric Profiling of Immune‐cell Phenotypes in co‐culture

Cells from co‐culture experiments (tumor cells with BMDCs, BMDMs, and/or splenic T cells) were gently resuspended and collected, pelleted (4°C, 2500 rpm, 5 min), and resuspended in staining buffer (PBS containing 0.5% BSA and 2 mM EDTA) to ∼1 × 10^6^ cells/mL.

For live/dead discrimination, cells were stained with Fixable Viability Stain 510 (BioLegend, 564406; 1 µL per sample) or Zombie viability dye (BioLegend, 423106) as indicated, for 15 min at room temperature in the dark, followed by two washes. Fc receptors were blocked using Mouse BD Fc Block (BioLegend, 553141; 1 µg per sample) for 15 min at 4°C.

Surface staining was performed using pre‐mixed antibody cocktails for 30 min at 4°C in the dark, followed by two washes. Antibodies used for immune phenotyping included:

Leukocyte and myeloid lineage markers: CD45 (BV510, BioLegend 567800; or APC/Cy7, BioLegend 561037), CD11b (Alexa Fluor 700, BioLegend 557960), F4/80 (BV650, BioLegend 743282).

Dendritic cell markers and activation: CD11c (PE/Cy7, BioLegend 561022; or BV605, BioLegend 563057), MHC‐II (I‐A/I‐E; PE, BioLegend 562010), CD80 (PerCP/Cy5.5, BioLegend 560526; or APC, BioLegend 560016), CD86 (PE, BioLegend 561963; or BV786, 740900).

Macrophage polarization marker: CD206 (Alexa Fluor 647, BioLegend 568809).

T‐cell markers: CD3ε (FITC, BioLegend 561827; or BB700, BioLegend 566495), CD4 (Alexa Fluor 488, BioLegend 569844), CD8a (PerCP, BioLegend 561092; or PE/Cy7, BioLegend 561097), CD25 (BV421, BioLegend 566228).

For intracellular staining, cells were fixed/permeabilized using a commercial fixation/permeabilization solution (per manufacturer's protocol), and stained with antibodies against: Foxp3 (PE CF594, BioLegend 567373) for Treg identification; IFN‐γ (PE, BioLegend 562020; or BV650, BioLegend 563854) for T‐cell effector function.

After staining, samples were filtered through a 300‐mesh nylon filter and acquired on a flow cytometer. Compensation was set using single‐stained controls; gating was performed on live singlets followed by CD45^+^ leukocytes, then lineage‐specific subsets (e.g., CD11c^+^ DCs; CD11b^+^F4/80^+^ macrophages; CD3^+^ T cells with CD4^+^/CD8^+^ subsets; Foxp3^+^ Tregs; IFN‐γ^+^ effectors). Full antibody details (fluorophore, vendor, catalog number, and working dilution) are provided in Supplementary Table S6.

#### 4.7.3 Flow Cytometric Profiling of Tumor‐infiltrating Immune Cells From Tumor Tissues

For tumor dissociation, a 5‐mL digestion cocktail was prepared per sample by mixing 10× collagenase/hyaluronidase (500 µL), DNase I (1 mg/mL; 750 µL), and RPMI‐1640 (3.75 mL). Orthotopic pancreatic tumors were excised, rinsed with sterile PBS, minced into fragments (≤2 mm), and incubated in 5 mL digestion cocktail at 37°C with shaking for 30 min.

Digested material was filtered through a 70‐µm cell strainer into a 50‐mL tube, and residual tissue was gently dissociated on the strainer using the rubber end of a syringe plunger. The strainer was rinsed with complete RPMI‐1640 and brought to 50 mL. Cells were pelleted (300 × g, 10 min, room temperature), resuspended to obtain a single‐cell suspension, and stained using the same viability, Fc‐blocking, surface, and intracellular staining workflows described above. Detailed information on the flow cytometry antibodies is provided in Supplementary Table S6.

### 4.8 Establishment of Murine Tumor Models

#### 4.8.1 Subcutaneous KPC A548 Tumor Model

Female C57BL/6 mice (8 weeks old) were shaved on the right lower dorsal flank and ear‐tagged prior to inoculation. KPC A548 cells were harvested, resuspended in sterile PBS at 2 × 10^7^ cells/mL, and kept on ice until injection. Mice were inoculated subcutaneously in the right dorsal flank with 2 × 10^6^ KPC A548 cells in 100 µL PBS using an insulin syringe. Tumor growth and body weight were monitored every 3 days using digital calipers. Mice were enrolled for treatment when the maximal tumor diameter exceeded 5 mm or tumor volume reached ≥100 mm^3^. Tumor volume was calculated as: *V* = (length × width × height)/2.

#### 4.8.2 Orthotopic KPC A548 Pancreatic Tumor Model

Female C57BL/6 mice and BALB/c nude mice (8 weeks old) were shaved on the left abdomen and ear‐tagged before surgery. KPC A548 cells were prepared as above (2 × 10^7^ cells/mL in sterile PBS) and maintained on ice. After disinfection of the left abdominal skin, the abdominal wall and peritoneum were carefully opened to expose the spleen and pancreas. The spleen/pancreas was gently exteriorized to facilitate injection, and 4 × 10^5^ KPC A548 cells in 20 µL PBS were injected into the pancreatic body using a 50‐µL microsyringe. The pancreas and spleen were returned to the abdominal cavity, and the peritoneum and skin were closed with absorbable sutures. Tumor progression and body weight were monitored, and mice were enrolled for treatment when the tumor diameter reached ∼5 mm (typically around day 8 post‐implantation). For luciferase‐expressing KPC A548 cells, tumor burden was additionally monitored by in vivo bioluminescence imaging.

#### 4.8.3 Orthotopic Pancreatic Cancer Liver Metastasis Model and Treatment

Orthotopic pancreatic tumors were established as described above. At the time of treatment enrollment, mice additionally received an intrasplenic injection of 5 × 10^5^ KPC A548 cells suspended in 25 µL PBS using a microsyringe to promote hepatic dissemination. Mice then underwent electroporation‐based ablation and other interventions according to the assigned treatment groups.

#### 4.8.4 Electroporation‐Based Ablation Treatment

Tumor‐bearing mice were randomly allocated to one of the following treatment arms: (i) Control, (ii) Nclx knockdown, (iii) electroporation (EP), and (iv) Nclx knockdown + EP; or, in the micelle‐combination experiments, (i) Control, (ii) CaCGP micelle mixture, (iii) EP, and (iv) EP + CaCGP.

EP ablation was performed using a two‐needle electrode array with a 5‐mm needle‐to‐needle spacing. Pulse parameters were set to an electric‐field strength of 1500 V/cm, pulse width of 100 µs, pulse frequency of 1 Hz, and 99 pulses. To improve field coverage, electroporation was delivered twice with the electrode rotated by 90° (i.e., two mutually orthogonal directions).

For the subcutaneous tumor model, mice were disinfected and anesthetized, and the two needles were inserted percutaneously into opposite sides of the tumor with the needles oriented parallel to the skin surface. EP was then delivered in two orthogonal directions as described above.

For the orthotopic pancreatic tumor model, mice were disinfected and anesthetized, and a ∼0.8‐cm incision was made in the left abdomen. The pancreas and spleen were gently exteriorized to fully expose the tumor. The two‐needle electrode was inserted across the tumor margins, and EP was delivered in two orthogonal directions. The pancreas and spleen were then returned to the abdominal cavity, and the peritoneum and skin were closed with absorbable sutures.

In combination groups, immediately after EP, CaCGP micelle mixture (100 µL) was administered by multi‐site intratumoral injection. The CaCGP mixture contained 100 mM CaCl_2_ and 6 mM CGP37157 (total CGP37157 content encapsulated in micelles). Control mice underwent sham procedures, in which the electrode was inserted and withdrawn without electroporation delivery.

After treatment, tumor size and body weight were monitored every 3 days.

#### 4.8.5 CD8^+^ T Cell Depletion Experiment

To investigate the functional contribution of CD8^+^ T cells to the antitumor efficacy of EP+CaCGP, CD8^+^ T cell depletion was performed in the orthotopic pancreatic tumor model. Briefly, mice received intraperitoneal injections of an anti‐mouse CD8α depleting antibody (200 µg per mouse; Selleck, Cat# A2102) starting on day 4 after orthotopic tumor implantation. Subsequently, mice were administered maintenance doses of 100 µg per mouse every other day until the experimental endpoint.

#### 4.8.6 Single‐cell RNA‐seq of Tumor Tissues and Downstream Analysis

Orthotopic pancreatic tumors were harvested on day 4 after treatment initiation, placed in tissue preservation solution, and transported on ice. Single‐cell RNA sequencing and primary data processing were outsourced to Wuhan Ruixing Biotechnology Co., Ltd. (Wuhan, China) using the BD Rhapsody platform. Briefly, fresh tumor tissues were enzymatically digested and mechanically dissociated into single‐cell suspensions, filtered through cell strainers, and subjected to quality control. Qualified suspensions were adjusted to an appropriate concentration and loaded onto BD Rhapsody microwell cartridges (“honeycomb” plates). Cell‐loading efficiency and capture‐bead occupancy were evaluated using the BD Rhapsody imaging system. Barcoded capture beads were added in excess to increase cell–bead co‐occupancy. After cell lysis, mRNA was captured on barcoded beads, and reverse transcription, cDNA synthesis, and library construction were performed according to the manufacturer's standard workflow.

Libraries were sequenced on an Illumina NovaSeq 6000 platform (paired‐end). After quality filtering, reads were processed using the BD Rhapsody Analysis Pipeline to demultiplex cell barcodes and UMIs, align reads to the reference genome, and generate gene‐by‐cell UMI count matrices. Downstream analyses were performed in R (version 4.4.2) and included removal of low‐quality cells, normalization, selection of highly variable genes, integration across experimental groups, dimensionality reduction, unsupervised clustering, and cell‐type annotation. Differences in cell‐type composition and within–cell‐type differential expression between groups were assessed, and results were visualized and functionally annotated as indicated. Cell–cell communication analyses were further conducted to compare intercellular signaling differences across treatment groups, primarily using Seurat (version 4.0) and CellChat.

Pseudotime analysis of CD8^+^ T‐cell subsets was performed using the Monocle3 package. CD8^+^ T cells were first extracted from the single‐cell transcriptomic dataset, followed by trajectory reconstruction and cell‐state ordering based on their transcriptomic profiles to evaluate the distribution of different CD8^+^ T‐cell subsets along the developmental/differentiation trajectory.

Immune‐cell functional states were scored using the AddModuleScore function in the Seurat package. Based on predefined function‐related gene sets, cytotoxicity scores in T cells, M1‐like inflammatory scores in macrophages, M2‐like immunosuppressive scores in macrophages, and maturation scores in dendritic cells were calculated. The gene sets used for scoring were as follows:

The T‐cell cytotoxicity gene set included: Gzmb, Prf1, Nkg7, Gzma, Ifng, and Ccl5. The macrophage M1‐like inflammatory gene set included: Il1b, Nos2, Tnf, Cxcl9, Cxcl10, and Cd86. The macrophage M2‐like immunosuppressive gene set included: Mrc1, Arg1, Chil3, Retnla, Il10, and Tgfb1. The dendritic‐cell maturation gene set included: Cd80, Cd86, Cd40, and Ccr7.

These functional scores were used to compare changes in the functional states of major immune‐cell subsets across treatment groups and to assist in evaluating treatment‐induced remodeling of the tumor immune microenvironment.

#### 4.8.7 Mouse Serum and Cell‐Culture Supernatant ELISA

For mouse serum, blood was collected by cardiac puncture under anesthesia on day 4 after treatment initiation. Whole blood was allowed to clot at room temperature for 2 h and centrifuged at 3,000 rpm for 15 min at 4°C. The supernatant was collected as serum and diluted 1:10 prior to cytokine measurement.

For cell‐culture supernatants, conditioned medium from KPC A548 cells was collected 24 h after the indicated treatments, centrifuged at 3,000 rpm for 15 min at 4°C to remove debris, and the clarified supernatant was used for analysis.

Cytokines were quantified using commercial ELISA kits following the manufacturers’ instructions (double‐antibody sandwich format). Standard curves were generated for each assay, and sample concentrations were calculated accordingly.

#### 4.8.8 Serum Biochemistry

Serum was prepared as described above, stored at −80°C, and analyzed in batch by Wuhan Servicebio (Wuhan, China). Biochemistry indices included alanine aminotransferase (ALT), aspartate aminotransferase (AST), creatinine, creatine kinase (CK), and amylase.

#### 4.8.9 Statistical Analyses

All statistical analyses were performed using GraphPad Prism 9 (GraphPad Software). Unless otherwise indicated, data are presented as mean ± s.e.m. (or mean ± s.d.), and figure legends specify the number of biological replicates (n) and the number of independent experiments. Comparisons between two groups were conducted using two‐tailed unpaired Student's t‐tests; when assumptions of normality or homoscedasticity were not met, Mann–Whitney U tests were applied, or data were appropriately transformed prior to analysis. Comparisons among three or more groups were performed using one‐way ANOVA followed by Tukey's post hoc multiple‐comparisons test; when variances were unequal, Welch's ANOVA with an appropriate multiple‐comparisons procedure was used; for nonparametric data, Kruskal–Wallis tests with Dunn's multiple‐comparisons correction were applied. For analyses involving two factors (e.g., treatment × time/dose), two‐way ANOVA was used; repeated‐measures data were analyzed using repeated‐measures ANOVA or mixed‐effects models, as appropriate, with multiple‐comparisons correction. Survival was analyzed using Kaplan–Meier curves and compared by the log‐rank (Mantel–Cox) test; when indicated, Cox proportional‐hazards regression was used to estimate hazard ratios with confidence intervals. All tests were two‐sided, and p < 0.05 was considered statistically significant. Significance is denoted as ns (not significant), *p < 0.05, **p < 0.01, ***p < 0.001, and ****p < 0.0001.

Animal sample sizes were determined based on preliminary experimental results, previous studies using similar PDAC animal models, and animal‐ethics considerations. After enrollment, animals were randomly assigned to experimental groups using a computer‐generated random‐number table, while ensuring comparable baseline tumor burden among groups as much as possible. Treatment procedures and subsequent data acquisition/analysis were performed by different investigators. Investigators responsible for tumor measurement, image acquisition, survival follow‐up, histopathological evaluation, and quantitative analysis of immunohistochemistry/immunofluorescence images were blinded to group allocation until data lock. Immunohistochemistry and immunofluorescence images were quantified independently by two blinded observers, and the average values were used for statistical analysis. Although a formal a priori power calculation was not performed at the initial study‐design stage, sample‐size estimation based on preliminary effect sizes indicated that 5–6 mice per group would provide approximately 80% power at α = 0.05 to detect the effect differences observed in the major efficacy endpoints of this study. All statistical tests were two‐sided, and p < 0.05 was considered statistically significant.

## Author Contributions

Experimental procedures were performed by **Chuanbing Zhao** and **Long He**. **Hongzhen Wei** and **Yu Lu** collected the clinical specimens. Chuanbing Zhao, **Zanglong Deng** and **Yunpeng Zhang** validated the experimental findings. Statistical analyses were conducted by Chuanbing Zhao and Yu Lu. The manuscript was written by Chuanbing Zhao, Yu Lu, and Long He. **Tao Yin** and **Ming Yang** supervised the study design and critically revised the manuscript.

## Funding

This study was supported by the National Natural Science Foundation of China (Number: 82472989; 82373227; 82173196), Hubei Provincial Natural Science Foundation General Program (Number: 2023AFB889), and Hubei Provincial Key Research and Development Project (Number: 2022BCA012).

## Ethical Statement

Informed consent was obtained from all study participants whose PDAC samples were used in this study. The study protocol involving human participants and specimens was approved by the Clinical Research Ethics Committee of Union Hospital, Tongji Medical College, Huazhong University of Science and Technology (Approval No. 20250476). All animal experiments were approved by the Animal Ethics Committee of Huazhong University of Science and Technology and were conducted at the Animal Centre of Huazhong University of Science and Technology in accordance with the approved institutional guidelines (Approval No. 20234505).

## Consent for Publication

All authors approved this manuscript for publication.

## Conflicts of Interest

The authors declare no conflicts of interest.

## Supporting information




**Supporting File**: advs78288‐sup‐0001‐SuppMat.docx.

## Data Availability

The data that has been published earlier and utilized in this research is appropriately referenced, whereas other raw datasets can be obtained from the corresponding authors with a reasonable request.
