# Supplementary material for: NCLX Inhibition plus Ca2+ Delivery Potentiates Electroporation and Antitumor Immunity in Pancreatic Ductal Adenocarcinoma
Source: Adv Sci (Weinh). 2026 Oct 25:e78288. Online ahead of print. doi: 10.1002/advs.78288 (PMC13696882; doi:10.1002/advs.78288)
Supplement: Supplementary file 1 [file ADVS-9999-e78288-s001.docx]

**Supplementary Materials**

**Supplementary Figures**

Figure S1

**Figure S1. Transient mitochondrial damage and calcium-dependent mitochondrial dysfunction following electroporation (EP).**

(A) Transmission electron microscopy (TEM) analysis of mitochondrial morphology in surviving tumor regions at different time points after IRE treatment (Day 1, Day 4, and Day 7). Mitochondrial swelling, cristae disruption, and matrix rarefaction were most pronounced on Day 1 post-IRE and gradually recovered by Day 7, indicating structural repair in surviving tumor cells. Arrowheads indicate representative damaged mitochondria.

(B) Cell viability of Panc-1 cells following exposure to increasing electric field intensities (0–800 V/cm). Quantification shows a significant reduction in viability at 600 V/cm and above, supporting the selection of 600 V/cm for subsequent in vitro experiments (n=6 per group).

(C-D) Gene set enrichment analysis (GSEA) showing enrichment of mitochondrial fusion (C) and mitochondrial organization (D) in EP -treated cells (EP vs Control).

(E, F) Mitochondrial calcium levels in KPC A548 cells after EP at different electric field strengths. Representative fluorescence images (E) and quantitative analysis (F) demonstrate a field strength–dependent increase in mitochondrial calcium accumulation (n=6 per group).

(G, H) Mitochondrial membrane potential in KPC A548 cells assessed by JC-1 staining after EP treatment. Representative images (G) and quantification of the JC-1 aggregate/monomer ratio (H) show a dose-dependent decrease in mitochondrial membrane potential with increasing electric field intensity (n=6 per group).

Data are presented as mean ± s.e.m. Statistical significance was assessed by two-tailed Student’s *t* test (two groups) and one-way ANOVA (multiple groups). **P < 0.05, ***P < 0.001; ****P < 0.0001.*

**Figure S2. Establishment of EP-resistant pancreatic cancer cells and validation of NCLX manipulation.**

(A) CCK-8 assay showing cell viability of parental Panc-1 cells (WT) and EP-resistant Panc-1 cells (Res) with or without electroporation (EP). EP-resistant cells exhibited significantly higher viability after EP compared with WT cells (n=6 per group).

(B) Representative colony formation images of WT and Res Panc-1 cells under non-treated (NT) or EP conditions (n=6 per group).

(C) Quantification of colony formation efficiency in (B). Res cells retained markedly higher clonogenic capacity after EP than WT cells (n=6 per group).

(D) Public dataset analysis (TCGA-PAAD and GTEx datasets) showing SLC8B1 (encoding NCLX) expression in pancreatic ductal adenocarcinoma (PAAD) tumor tissues compared with normal pancreatic tissues.

(E) Immunoblot analysis of NCLX protein levels in Panc-1 cells transfected with three independent siRNAs targeting NCLX (si#1–si#3) or negative control (NC); β-Actin served as a loading control (n=3 per group).

(F) Densitometric quantification of NCLX protein levels in (E), normalized to β-Actin and presented relative to NC (n=3 per group).

(G) CCK-8 assay showing that NCLX knockdown (Res+si#1) reduced the post-EP viability of EP-resistant Panc-1 cells compared with Res control cells (n=6 per group).

(H) Immunoblot validation of Nclx knockout efficiency in three independent Nclx- knockdown clones (KO#1–KO#3) versus negative control (NC) in KPC-A548; β-Actin served as a loading control.

(I) Immunoblot validation of Nclx overexpression (OE) compared with negative control (NC) in Panc-1 cells; β-Actin served as a loading control.

Data are presented as mean ± s.e.m. Statistical significance was assessed by two-tailed Student’s *t* test (two groups) and one-way ANOVA (multiple groups).*; **P < 0.01; ****P < 0.0001;*

**Figure S3: Additional validation of pyroptosis activation following NCLX knockdown and EP treatment.**

(A–D) Quantitative analysis of western blotting (WB) for pyroptosis-related markers in PANC-1 cells across different groups, corresponding to Figure 2S (n=3 per group).
(E–H) Quantitative analysis of western blotting (WB) for pyroptosis-related markers in KPC cells across different groups, corresponding to Figure 2S (n=3 per group).
(I–K) Representative IHC images and quantitative analysis of NCLX, Ki67, and cleaved-CASP1 in the Control, EP, shNCLX, and EP+shNCLX groups (n=5 per group).

Data are presented as mean ± s.e.m. Statistical significance was assessed by two-tailed Student’s *t* test (two groups) and one-way ANOVA (multiple groups). **P < 0.05; **P < 0.01; ***P < 0.001; ****P < 0.0001.*

**Figure S4. Induction of mitochondrial Ca²⁺ overload by combining EP with NCLX inhibition and exogenous Ca²⁺ delivery enhances tumor cell killing.**

(A, B) Dose–response curves showing the effects of CGP37157 (A) or extracellular Ca²⁺ (B) on Panc-1 cell viability under non-electroporation conditions. CGP37157 or Ca²⁺ alone exhibited minimal cytotoxicity within commonly used concentration ranges, with evident inhibition only at high doses (n=6 per group).

(C, D) Dose–response curves showing the effects of CGP37157 (C) or extracellular Ca²⁺ (D) on KPC A548 cell viability under non-electroporation conditions (n=6 per group).

(E, F) Heatmap summary of cell viability after EP in Panc-1 (E) and KPC A548 (F) cells treated with increasing concentrations of CGP37157 and Ca²⁺ immediately after EP. Combined treatment led to a dose-dependent reduction in viability that was markedly stronger than either treatment alone (n=6 per group).

(G, H) Cell viability of Panc-1 and KPC A548 cells following the indicated treatments. EP refers to electroporation; CGP refers to mPEG-PCL@CGP37157 micelles. Amlodipine was dissolved in DMSO and applied to pancreatic cancer cells at a final concentration of 10 μM (n=6 per group).

(I) Representative colony formation images and quantification of Panc-1 cells following the indicated treatments, showing enhanced inhibition of colony formation with EP+CaCGP (n=6 per group).
(J) Representative colony formation images and quantification of KPC A548 cells following the indicated treatments, showing enhanced inhibition of colony formation with EP+CaCGP (n=6 per group).

Data are presented as mean ± s.e.m. Statistical significance was assessed by two-tailed Student’s *t* test (two groups) and one-way ANOVA (multiple groups). *****P < 0.0001; ns, not significant.*

**Figure S5 | EP+CaCGP induces mitochondrial dysfunction, pyroptotic cell death, and pyroptosis-dependent cytotoxicity in KPC A548 cells.**
(A,B) Representative Rhod-2 AM fluorescence images and quantification showing mitochondrial Ca²⁺ accumulation in KPC A548 cells after the indicated treatments (Control, CGP, EP, EP+CGP, EP+CaCGP) (n = 6 per group).
(C,D) Representative JC-1 staining and quantification of mitochondrial membrane potential (JC-1 aggregates/monomers) in KPC A548 cells under the indicated conditions (n = 6 per group).
(E,F) Representative DCFH-DA fluorescence images and quantification of intracellular ROS levels in KPC A548 cells after the indicated treatments (n = 6 per group).
(G) Representative bright-field images showing pyroptosis-like morphological changes (arrowheads) in KPC A548 cells following the indicated treatments.
(H) Extracellular ATP release in KPC A548 cells after the indicated treatments (n = 3 per group).
(I) LDH release in KPC A548 cells after the indicated treatments (n = 3 per group).
(J) Representative immunofluorescence images showing calreticulin (CRT) exposure on the cell surface after the indicated treatments in KPC A548 cells.
(K,L) Representative Annexin V-FITC/PI flow cytometry plots and quantification of Annexin V⁺/PI⁺ cells in KPC A548 cells after the indicated treatments (n = 3 per group).
Data are presented as mean ± s.e.m. Statistical significance was assessed by two-tailed Student’s *t* test (two groups) and one-way ANOVA (multiple groups). ****P < 0.001; ****P < 0.0001.*

**Figure S6: EP+CaCGP promotes activation of the NF-κB/NLRP3/CASP1/GSDMD pyroptosis pathway in pancreatic cancer cells.**

(A) Quantitative analysis of western blotting (WB) for pyroptosis-related markers (N-GSDMD, p-P65, NLRP3, and cleaved CASP1) in PANC-1 cells under the indicated conditions, corresponding to Figure 4M (n=3 per group).

(B-C) Western blot analysis and quantitative of pyroptosis-associated markers (N-GSDMD, p-P65, NLRP3, and cleaved CASP1) under the indicated conditions in KPC-A548 (n = 3 per group).

Data are presented as mean ± s.e.m. Statistical significance was assessed by two-tailed Student’s *t* test (two groups) and one-way ANOVA (multiple groups). **P < 0.05; **P < 0.01; ***P < 0.001; ****P < 0.0001; ns, not significant.*

**Figure S7: EP+CaCGP induces mitochondrial Ca²⁺ overload-dependent pyroptosis in pancreatic cancer cells.**

(A) Quantitative analysis of western blotting (WB) for pyroptosis-related markers in PANC-1 cells across different groups, corresponding to Figure 4N (n=3 per group).

(B-C) Western blot analysis and quantitative of pyroptosis-associated markers, including N-GSDMD and cleaved CASP1, in KPC A548 cells treated with EP+CaCGP with or without MCU-i11 (n = 3 per group).

(D) CCK8 analysis under the indicated conditions (Control, EP, EP+CaCGP, shNLRP3, shGSDMD, EP+CaCGP+ shNLRP3, EP+CaCGP+ shGSDMD, EP+CaCGP+ shGSDMD+shNLRP3) in KPC-A548 (n = 6 per group).

Data are presented as mean ± s.e.m. Statistical significance was assessed by two-tailed Student’s *t* test (two groups) and one-way ANOVA (multiple groups). *****P < 0.0001;*

**Figure S8: IHC analysis showing that EP+CaCGP inhibits pancreatic cancer cell proliferation and promotes pyroptosis**

(A) Representative Ki-67 IHC images and quantification in the indicated groups (n=5 per group). (B) Representative cleaved-CASP1 IHC images and quantification in the indicated groups (n=5 per group).

Data are presented as mean ± s.e.m. Statistical significance was assessed by two-tailed Student’s *t* test (two groups) and one-way ANOVA (multiple groups). *****P < 0.0001;*

**Figure S9: NCLX inhibition synergizes with EP to enhance local effector immunity within tumors**

(A) Representative multicolor immunofluorescence images of tumor sections from the indicated treatment groups, showing CD8⁺ T cells (including IFN-γ⁺ CD8⁺ subsets), dendritic cells (DCs) and DC maturation (CD86), and macrophage polarization markers (M1-/M2-like features; markers as indicated) (scale bar, 10 μm).
(B) Quantification of multicolor immunofluorescence staining in (A), including total CD8⁺ T cells, IFN-γ⁺ CD8⁺ T cells, DC abundance and the proportion of CD86⁺ mature DCs, as well as macrophage polarization toward M1-like versus M2-like phenotypes (n = 3 per group).
Data are presented as mean ± s.e.m. Statistical significance was assessed by two-tailed Student’s *t* test (two groups) and one-way ANOVA (multiple groups). *****P < 0.0001.*

**Figure S10. EP+CaCGP promotes CD8⁺ T-cell state transition and strengthens immune-cell communication within the TME.**
(A) Pseudotime distribution of CD8⁺ T-cell subsets, showing that naive CD8⁺ T cells are mainly localized at the early stage of pseudotime, activated and effector CD8⁺ T cells are enriched in the middle stage, and cytotoxic CD8⁺ T cells tend to localize to the late stage.

(B,C) CellChat-based global cell–cell communication analysis showing the number of interactions between macrophages and T cells (B) and between dendritic cells and T cells (C) after the indicated treatments.

**Figure S11: The gating strategy for flow cytometry of PDAC tumor tissues.**

**Figure S12: CD8⁺ T-cell immunity contributes to EP+CaCGP-mediated tumor control and systemic antitumor effects**

(A-B) Representative IHC images and quantitative analysis of Ki-67 and cleaved CASP1 staining in nude mouse orthotopic tumor models from the indicated groups (n=5 per group).

(C) Kaplan–Meier survival curves of mice bearing tumors under the indicated treatment conditions, showing that CD8⁺ T-cell depletion markedly reduces the survival benefit induced by EP+CaCGP (n = 6 per group).

(D) Tumor growth curves of subcutaneous tumors measured on days 0, 3, 6, 9, and 12 after enrollment in mice from the indicated groups in the abscopal effect model (n=6 per group).

Data are presented as mean ± s.e.m. Statistical significance was assessed using the log-rank (Mantel–Cox) test, two-tailed Student’s t test, or one-way ANOVA. **P* < 0.05; ****P* < 0.001; *****P* < 0.0001; *ns*, not significant.

**Figure S13: EP+CaCGP induces pyroptosis-associated marker expression, and CASP1 knockdown attenuates its survival benefit in vivo.**

(A) cleaved Caspase-1 staining and quantification of cleaved Caspase-1⁺ cells (%). EP refers to electroporation; CGP refers to mPEG-PCL@CGP37157 micelles; Ca refers to exogenous Ca²⁺ supplementation; CaCGP refers to the combined local delivery of Ca²⁺ and CGP37157-loaded micelles; and EP+CaCGP refers to electroporation followed by local co-delivery of Ca²⁺ and CGP37157-loaded micelles (n=6 per group; scale bar = 50 um).

(B) N-GSDMD staining and quantification of N-GSDMD⁺ cells (%) (n = 6 per group; scale bar = 50 um).

(C) Kaplan–Meier survival curves of mice bearing control or CASP1-knockdown KPC A548 tumors under EP+CaCGP treatment, showing that CASP1 knockdown attenuates the survival benefit induced by EP+CaCGP (n = 6 per group).

(D-G) Representative IHC images and quantitative analysis of Ki-67 and cleaved CASP1 staining in C57BL/6 orthotopic tumor models from the indicated groups (n=6 per group; scale bar = 50 um).

Data are presented as mean ± s.e.m. Statistical significance was assessed using one-way ANOVA or Wilcoxon rank-sum test, as appropriate. **P* < 0.05; ***P* < 0.01; ****P* < 0.001; *****P* < 0.0001; *ns*, not significant.

**Figure S14 | Single-cell transcriptomic analysis and immunofluorescence validation of SPP1–CD44 signaling and macrophage polarization.**

(A,B) Single-cell transcriptomic analysis showing SPP1 expression in ductal cells (A) and CD44 expression in macrophages (B) in the indicated groups, visualized by UMAP clustering.
(C) Representative multicolor immunofluorescence images showing the polarization of macrophages (F4/80⁺CD86⁺CD206⁺) in tumors from control, EP, EP+CaCGP, and EP+CaCGP+OE-Spp1-treated mice; Quantification of F4/80⁺CD86⁺ macrophages (M1-like) among F4/80⁺ cells per high-power field (HPF) from multicolor immunofluorescence images (n = 3 per group).

Data are presented as mean ± s.e.m. Statistical significance was assessed by two-tailed Student’s *t* test (two groups) and one-way ANOVA (multiple groups). ****P < 0.001; ****P < 0.0001;*

**Table S1: Antibodies used in western blot, immunohistochemistry, and immunofluorescence**

| **Antibody** | **Source** | **IDENTIFIER** |
| --- | --- | --- |
| SLC8B1 Rabbit Polyclonal Antibody | Abiowell | AWA70579 |
| NLRP3 Rabbit mAb | Abclonal | A24294 |
| Cleaved GSDMD (N Terminal) Rabbit mAb | Abclonal | A22523 |
| Rabbit polyclonal antibody to GSDMD | Affinity | AF4012 |
| Cleaved-Caspase 1 (Asp296), p20 Antibody | Affinity | AF4005 |
| Ki67 Rabbit mAb | Abclonal | A20018 |
| Calreticulin Rabbit pAb | Abclonal | A1066 |
| Phospho-NF-kB p65/RelA-S536 Rabbit pAb | Abclonal | AP0475 |
| HRP, Goat Anti-Rabbit IgG | Abbkine | A21020 |
| Cy3 conjugated Goat Anti-Rabbit IgG (H+L) | Servicebio | GB21303 |
| β-actin Rabbit mAb | Servicebio | GB15003 |
| CD8a Monoclonal antibody | proteintech | Cat#66868-1-Ig |
| IFN-gamma Polyclonal antibody | proteintech | Cat#15365-1-AP |
| F4/80 Polyclonal antibody | proteintech | Cat# 28463-1-AP |
| Anti-B7-2/CD86 Antibody | BOSTER | Cat#BM4121 |
| Anti-CD206/MRC1 Antibody | BOSTER | Cat#A02285-2 |
| Anti-CD11c/Integrin Alpha X/ITGAX Antibody | BOSTER | Cat# A00357-1 |

**Table S2. Reagents, kits and material information**

| **REAGENT or RESOURCE** | **SOURCE** | **IDENTIFIER** |
| --- | --- | --- |
| DMEM cell culture  medium | GIBCO | Cat#11995-065 |
| RPMI 1640 cell culture  medium | GIBCO | Cat#61870036 |
| DMEM/F-12 Gibco™ | GIBCO | Cat#11320033 |
| Penicillin/streptomycin |  | Cat#15140-122 |
| Fetal bovine serum | Corning | Cat#35-010-CV |
| Caspase-1 Inhibitor II | AmBeed | Cat# A1329237 |
| DMSO | Sigma | Cat#67-68-5 |
| DAPI | Solarbia | Cat# C006 |
| Protease inhibitor  cocktail | Beyotime | Cat#P1005 |
| Phosphatase Inhibitor Cocktail I (100× in DMSO) | MedChemExpress | HY-K0021 |
| Phosphatase Inhibitor Cocktail Ⅱ (100× in ddH2O) | MedChemExpress | HY-K0022 |
| TRIzol | TransGen Biotech | Cat#ET111-01-V2 |
| Recombinant Mouse Protein Spp1 | MedChemExpress | Cat#HY-P78358 |
| BCA Protein Assay Kit | Beyotime | Cat#P0012S |
| Lipofectamine™ 8000 | Beyotime | Cat#C0533 |
| WesternBright ECL | Advansta | Cat#K-12045-D50 |
| FastPure Cell/Tissue  Total RNA isolution Kit  V2 | Vazyme | Cat#RC112-01 |
| Super ECL Detection  Reagent ECL | Yeasen | Cat#36208ES76 |
| TransStart® Top Green  qPCR SuperMix (+Dye  II) | TransGen Biotech | Cat# AQ132-21 |
| TransStart Top Green  qPCR SuperMix | TransGen Biotech | Cat#AQ132-21 |
| SDS-PAGE Sample Loading Buffer, 5× | Seven | Cat#SW116-01 |
| Prestained Protein Marker | YAMAY BIOTECH | Cat#WJ102 |
| Prestained Protein Marker | Servicebio | Cat#G2085-250UL |
| Mouse IFN-γ ELISA Kit | JONLNBIO | Cat# JL10967 |
| Mouse IL-6 ELISA Kit | JONLNBIO | Cat# JL20268 |
| Mouse TNF-α ELISA Kit | JONLNBIO | Cat# JL10484 |
| Mouse IL-12 ELISA Kit | JONLNBIO | Cat# JL20246 |
| EasySep™ Mouse T Cell Isolation Kit | STEMCELL | Cat# 19851 |
| Enhanced ATP Assay Kit | Beyotime | Cat# S0027 |
| BD Cytofix/Cytoperm Fixation/Permeabilization Kit | BD | Cat#554714 |
| Pancreatic Cancer Organoid Kit | DAXIANG | Cat#OK100118 |
| 0.45 μM NC membrane | Amershan protran | Cat#10600002 |
|  |  |  |

**Table S3. PCR primers**

| **name** | **Sense or antisense** | **Sequence (5’ to 3’)** |
| --- | --- | --- |
| Human-SLC8B1 | sense | CCAGGGACCTGGAAGGCT |
|  | antisense | GCTCCGGTGGCCTCG |
| Mouse-Slc8b1 | sense | GTGTCACCTTCCTGGCCTTT |
|  | antisense | TGACCAGCACCCCAAACAGA |
| ACTB#1 | sense | CATGTACGTTGCTATCCAGGC |
|  | antisense | CTCCTTAATGTCACGCACGAT |
| Human-SPP1 | sense | TTTGCCTCCTAGGCATCACC |
|  | antisense | GGCCACAGCATCTGGGTATT |
| Mouse-SPP1 | sense | AGCAAGAAACTCTTCCAAGCAA |
|  | antisense | GTGAGATTCGTCAGATTCATCCG |

**Table S4. siRNA and shRNA information**

| name | Sequence |
| --- | --- |
| SLC8B1-siRNA | CUAUGGUGUCUAUGAGAUATT |
| NLRP3-shRNA | GUGCGUUAGAAACACUUCATT |
| Nlrp3-shRNA | GGUGAAAUGUACUUAAAUCTT |
| GSDMD-shRNA | CGUGGUGACUGAGGUGCUATT |
| Gsdmd-shRNA | GGAUAUCCUUCUCGUCUCATT |

**Table S5. Lentiviral vector information**

| name | Sequence |
| --- | --- |
| LV-Slc8b1-RNAi | CCAGACATCTTCAGTGCTTTA |
| LV-Casp1-RNAi | GGTATCCAGGAGGGAATATGT |

**Table S6: Flow cytometry antibodies**

| **Antibody** | **Source** | **IDENTIFIER** |
| --- | --- | --- |
| Annexin V-FITC/PI | Servicebio | G1511-100T |
| PE/Cy7 anti-mouse CD11c | Biolegend | 561022 |
| Alexa Fluor647 anti-mouse CD206 | Biolegend | 568809 |
| BV421 anti-mouse CD25 | Biolegend | 566228 |
| BV786 anti-mouse CD86 | Biolegend | 740900 |
| FITC anti-mouse CD3ε | Biolegend | 561827 |
| Alexa Fluor700 anti-mouse CD11b | Biolegend | 557960 |
| BV510 anti-mouse CD45 | Biolegend | 567800 |
| PerCP/Cy5.5 anti-mouse CD80 | Biolegend | 560526 |
| PE anti-mouse CD86 | Biolegend | 561963 |
| PerCP anti-mouse CD8a | Biolegend | 561092 |
| PE anti-mouse IFNγ | Biolegend | 562020 |
| PE anti-mouse I-A/I-E | Biolegend | 562010 |
| Zombie | Biolegend | 423106 |
| APC anti-mouse CD80 | Biolegend | 560016 |
| BB700 Anti-Mouse CD3e | Biolegend | 566495 |
| BV605 anti-mouse CD11c | Biolegend | 563057 |
| BV650 anti-mouse F4/80 | Biolegend | 743282 |
| PE/Cy7 anti-mouse CD8a | Biolegend | 561097 |
| Alexa Fluor 488 anti-mouse CD4 | Biolegend | 569844 |
| APC Cy7 anti-mouse CD45 | Biolegend | 561037 |
| PE CF594 anti-mouse Foxp3 | Biolegend | 567373 |
| BV 650 anti-mouse IFN-γ | Biolegend | 563854 |
| Mouse BD Fc Block | Biolegend | 553141 |
| Fixable Viability Stain 510 | Biolegend | 564406 |
